# Cognitive dysfunction in mice lacking proper glucocorticoid receptor dimerization

**DOI:** 10.1371/journal.pone.0226753

**Published:** 2019-12-23

**Authors:** Kelly Van Looveren, Michiel Van Boxelaere, Zsuzsanna Callaerts-Vegh, Claude Libert

**Affiliations:** 1 Center for Inflammation Research, VIB, Ghent, Belgium; 2 Department of Biomedical Molecular Biology, Ghent University, Ghent, Belgium; 3 Laboratory of Biological Psychology, KULeuven, Leuven Belgium; 4 Leuven Research Institute for Neuroscience & Disease (LIND), Leuven, Belgium; 5 mINT Mouse Behavioural Core Facility, KULeuven, Leuven, Belgium; Technion Israel Institute of Technology, ISRAEL

## Abstract

Stress is a major risk factor for depression and anxiety. One of the effects of stress is the (over-) activation of the hypothalamic-pituitary-adrenal (HPA) axis and the release of stress hormones such as glucocorticoids (GCs). Chronically increased stress hormone levels have been shown to have detrimental effects on neuronal networks by inhibiting neurotrophic processes particularly in the hippocampus proper. Centrally, GCs modulate metabolic as well as behavioural processes by activating two classes of corticoid receptors, high-affinity mineralocorticoid receptors (MR) and low-affinity glucocorticoid receptors (GR). Upon activation, GR can modulate gene transcription either as a monomeric protein, or as a dimer interacting directly with DNA. GR can also modulate cellular processes via non-genomic mechanisms, for example via a GPCR-protein interaction. We evaluated the behavioral phenotype in mice with a targeted mutation in the GR in a FVB/NJ background. In GR^dim/dim^ mice, GR proteins form poor homodimers, while the GR monomer remains intact. We evaluated the effect of poor GR dimerization on hippocampus-dependent cognition as well as on exploration and emotional behavior under baseline and chronically increased stress hormone levels. We found that GR^dim/dim^ mice did not behave differently from GR^wt/wt^ littermates under baseline conditions. However, after chronic elevation of stress hormone levels, GR^dim/dim^ mice displayed a significant impairment in hippocampus-dependent memory compared to GR^wt/wt^ mice, which correlated with differential expression of hippocampal *Bdnf/TrkB* and *Fkbp5*.

## Introduction

Stress is commonly defined as a state of real or perceived threat to homeostasis [1]. Short-term stress serves as an important mechanism to survive, while chronic stress is detrimental and an important contributor to major psychiatric disorders such as depression and anxiety [2]. In the presence of stressors, the hypothalamic-pituitary-adrenal (HPA) axis is activated and initiates a complex range of responses involving the endocrine, nervous and immune system, collectively known as the stress response [3, 4]. Centrally, chronic elevation of the stress response will affect neuronal functioning by inhibiting neurogenesis, proper synaptic pruning and changes in neurotransmitter systems [5, 6], and induce a wide range of behavioral changes including cognitive processes.

One of the hormones released in response to stress, are glucocorticoids (GCs). The physiological functions of GCs include regulation of metabolic homeostasis, immune function, cell proliferation and survival, and behaviour including cognition [7–9], while endogenous excess or dysregulation of GCs is associated with mood changes, including depression and hypomanic symptomatology [10].

GCs exert their effects via two receptors: the mineralocorticoid receptor (MR) and the glucocorticoid receptor (GR), which differ in their affinity and regional distribution in the brain. The MR has a more restricted distribution with high expression in the hippocampus and lower levels in cortical layers and the amygdala [11]. GR is expressed much more widely and is found in high concentrations in the hippocampus, aminergic neurons in the brainstem and in nuclei that are part of the HPA-axis, such as the hypothalamic PVN [12, 13]. Due to their difference in affinity for GCs, MRs are substantially activated by low levels of GCs, such as those circulating at rest, whereas lower-affinity GR becomes fully occupied only when there are high levels of hormone, such as during stress exposure and at a circadian peak and by pharmacological treatment with GCs [12]. GRs exert their action either as monomers or as homodimers by directly influencing DNA transcription. In addition, GCs can exert fast non-genomic effects, for example by interacting with membrane-bound GR proteins [14–16].

Patients suffering from major depression disorder (MDD) have been linked to dysfunction in the HPA axis [17], reduced sensitivity and deregulated feed-back mechanisms to GC [18]. While mutations within the ligand binding domain of GR gene can lead to a complex endocrinological phenotype, typified as ‘GC resistance syndrome’ [19], polymorphic variations might account for more subtle changes in GR function and indeed have been associated with MDD [20]. Cognitive aspects of depression mostly aggregate around two main foci: cognitive biases (‘negative thinking’) and cognitive deficits, such as impaired attention and concentration, short-term memory, autobiographical memory specificity and executive function [21–23]. The hippocampus has been attributed a major role in the cognitive impairment observed in MDD, due to its general central role in learning and memory [24–26], and the structural changes observed in the hippocampus proper in patients with MDD. These changes are direct or indirect consequences of excessive circulating GC [27–29] affecting neurotrophic processes and synaptic pruning. Furthermore, improving hippocampal neurogenesis has been proposed to be a promising therapeutic avenue for MDD [30]. In addition, dysfunction in dopamine signaling and in the salience network have been associated with MDD [31] and more specifically with impaired specificity of autobiographical memory [32–36]. In mice, inhibition of dopaminergic signaling or inactivation of dopaminergic receptors in the hippocampus induced depression-like behavioral phenotypes and impaired cognition [37–45].

We made use of the GR^dim/dim^ mutant mouse [46] with a point mutation (A458T) in the DNA binding domain (DBD) of the GR, by which it forms much weaker homodimers and less robust DNA binding, while leaving the monomeric GR function intact [46, 47]. The GR^dim/dim^ mouse model has proven important to dissect mechanistic pathways employed by GR in controlling (patho-)physiological phenomena. We compared the cognitive phenotype of GR^dim/dim^ mutant mice to their wildtype litter mates under baseline and under chronic stress hormone levels.

Under baseline conditions we found no effect of the mutation. By contrast, we identified impairment in hippocampus-dependent memory in these mice under chronic stress condition implicating monomer-dependent mechanisms in GR regulation of memory.

## Materials and methods

### Animals and reagents

GR^dim/dim^ mice were generated by Reichardt et al. and kept on an FVB/NJ background [46]. The mice were made available by Prof. Jan Tuckermann, Ulm University, Germany, and bred in our laboratories. Heterozygous GR^dim/wt^ mice were intercrossed to generate GR^wt/wt^ and GR^dim/dim^ homozygous mutant mice. All offspring was genotyped by PCR on genomic DNA isolated from toe biopsies. Mice were bred in IRC/VIB Ghent and kept in individually ventilated cages under a 12-hour dark/light cycle in a conventional facility. For behavioural testing, mice were transported to KUL. All mice were 9 weeks old on the first day of testing. We included n = 12 GR^wt/wt^ and n = 11 GR^dim/dim^ animals in the testing battery. 4 animals died after baseline testing (n = 3 GR^wt/wt^ and n = 1 GR^dim/dim^ animals) probably due to seizures [48]. All behavioural testing was performed during the light phase of the activity cycle, with the exception of the 23h-activity test. We used females only, since stress has clear sex-specific effects and males have been shown to be more resistant to stress [49, 50]. In addition, female animals were included due to the propensity of additional stress due to hierarchical fighting in co-housing male mice. All animal experiments were approved by the animal ethical committee of University of Leuven.

### Reagents

Corticosterone (CORT) (27840, Sigma-Aldrich NV) was dissolved in b-cyclodextrin solution (4% in tap water, C4767, Sigma-Aldrich NV) to a concentration of 36.5 mg/L [51] and provided in drinking water over 4 weeks. This dose has been shown to induce depression and anxiety-like features in different mouse strains including blunting of corticosterone response following an acute stressor [51, 52], while increasing the corticosterone levels less than what is observed after acute or chronic stress exposure [53–55]. Treatment was maintained during behavioural testing to prevent rebound effects. At the end of the behavioural tests, dorsal hippocampus was isolated and snap frozen.

### Timeline of behavioural testing and corticosterone treatment

**Fig 1:**
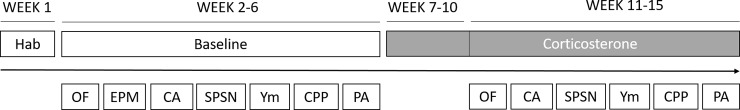
Order of tests. Open field (OP), elevated plus maze (EPM), 23h cage activity (CA), anhedonia (AnH), social preference and social novelty (SPSN), Y-maze spontaneous alternations (Ym), conditioned place preference (CPP), passive avoidance (PA).

After one week of habituation (week 1), we first tested all animals under baseline conditions (week 2–6), and then subjected them to chronic corticosterone treatment for 4 weeks (week 7–10) and testing (week 11–15)(see Fig 1). This schedule allowed for comparison of behaviour under two conditions within one animal and provided insight into corticosterone effects. In between the two blocks of behavioural testing, we provided a testing interval of 4 weeks during which the CORT was administered. Minimal handling was done to the animal. The order of tests was as follows: open field (OF), elevated plus maze (EPM), 23h cage activity (CA), anhedonia (AnH), social preference and social novelty (SPSN), Y-maze spontaneous alternations (Ym), conditioned place preference (CPP), passive avoidance (PA). EPM testing during CORT treatment was impossible, because the mice continuously jumped off the setup.

### Explorative and emotional behaviour

*Spontaneous cage activity* (CA) was recorded over a period of 20 hours in a home cage [56]. This test provides insight into general exploration, changes in diurnal pattern or nocturnal activity, motor impairment, and general arousal. To measure CA, mice were placed individually in transparent cages (26.7 cm x 20.7 cm) located between three IR photo beams connected to a computerized activity logger. Activity was registered as the number of beam crossings for each 30 min interval, during a 20 h recording period. Following a 15 min habituation, registration of beam crossings started at 4:30 pm during the pre-UCMS activity test and at 4:30 pm during the post-UCMS activity test, with lights being switched off at 7 pm (12h on/off cycle). Cage activity during the dark phase of the recording period was analysed.

In the *open field* (OF), we measured locomotor activity, exploratory and emotional behaviour as previously described [56]. After a 30-minute dark-adaption period, mice were placed in the brightly lit open field area (50 x 50cm^2^). After 1 min of habituation, exploratory behaviour was recorded for 10 min using an automated video tracking system (ANY-maze^™^ Video Tracking, Stoelting Co. IL, USA). Variables analysed were distance travelled and time spent in the centre or corner of the open field arena. Centre exploration is usually considered to reflect conflict resolution, while continuing wall-hugging (thigmotaxis) is a behaviour observed in conditions that evoke anxious behaviour.

In the *elevated plus maze* (EPM), we assessed anxiety-related exploration [56]. In contrast to the open field, the elevated plus maze provides closed arms, which are the preferred zone for mice. Venturing onto the open and elevated arms is challenging for mice and anxious animals will avoid the open space entirely. Mice were placed in the centre of a plus-shaped maze, consisting of two open arms (5 cm wide) without walls and two arms closed by side walls. Anxiety-related exploration was recorded for 11 min (1 min habituation and 10 min recording) by five IR photo beams connected to a computerized activity logger. There is one IR photo beam at the entry of each arm. One IR beam records the relative time spent in the open arm.

### Social behaviour and cognition

*Social approach and social memory* (SPSN) was assessed in a 3-compartment setup described previously [57]. In short, the setup consisted of a central start box that connected via a manual guillotine door to 2-side boxes containing small cylindrical holding cages. The holding cages could contain one stranger mouse. Exploration was recorded using an overhead webcam and an automated video tracking system (ANY-maze^™^ Video Tracking, Stoelting Co. IL, USA). The test entailed 3 phases. During acclimation, mice were placed in the central chamber for 5 min to habituate to the setup. During this habituation phase, access to the side chambers was prevented by closing the guillotine doors. After 5 min, a stranger mouse was introduced in one of the cylindrical cages on either left or right side (side was counter balanced). Approach behaviour towards the stranger 1 mouse (distance of nose < 2 cm) and preference towards this mouse over the empty wire cage was recorded for 10 min as a measure of sociability. After 10 min, a second stranger mouse (from a different cage, but same sex), was introduced on the side of the empty wire cage. Again, approach behaviour was measured for 10 min, and preference for stranger 2 over stranger 1 was considered a readout for social novelty and social memory. Parameters such as path length, walking speed and time spent close to the wire cages were extracted from ANYmaze tracking software.

*Spontaneous alternations in the Y-maze* was assessed as a readout of working memory function [58] as well as responsiveness to novelty [58, 59]. The Y-maze consisted of 3 arms (5cm wide, 30cm long and enclosed by 30 cm high wall made of grey plastic) connected by a central small area (diameter 5 cm). Mice were placed in the centre for 10 min exploration of all arms. Locomotion was recorded by a webcam connected to a PC. Entries into all arms were noted (all 4 paws had to be inside the arm for a valid entry) and an alternation was counted if an animal entered three different arms consecutively (i.e. 1-2-3, 2-3-1, 3-2-1). Percentage spontaneous alternation (%) was calculated according to following formula: [(number of alternations)/(total number of arm entries-2)]x100 [60, 61].

*Novel object recognition* was used to evaluate the innate responsiveness to novel objects. Mice were placed individually in the open field setup for 10 min. After 10 min, 2 identical objects were placed inside the box and time to explore (approach nose distance < 2cm from object) was recorded. 24h later, the animals were placed back in the open field, two new objects (one looking identical and one novel) were placed back in the box, and approach time and preference for novel object was recorded.

*Conditioned place preference* assesses the ability of an animal to distinguish and remember a specific context that was associated with a reward [62, 63]. Based on lesion studies, several brain regions such as amygdala, fornix, prefrontal cortex, and hippocampus affect conditioned place preference [63–65]. The place preference box was a three-compartment box, 2 large compartments of 30 x 30cm and a small (10 x 10cm) start compartment in between. The large compartments differed in their contextual configurations (tactile and olfactory dimensions). One context was consistently associated with a reward, while the other context was not. After habituation the animals to the reward pellets (in a neutral context) and to the setup (2 x 10min /day), mice were food conditioned to one of the contexts. During conditioning trials (10 min twice a day) mice are placed in the central start box and time to retrieve the pellet (latency to reward) and number of errors (entering wrong context) are recorded. After 6 trials, mice are again placed in the start box and time spent in each context was recorded in the absence of a food reward. Preference for food paired context reflects robust contextual memory.

To assess hippocampus-dependent contextual fear memory retention, animals were tested in the *passive avoidance* test [66–70]. Dark-adapted mice were placed in a brightly lit chamber with access to a dark box. Upon entering the dark box, the access is closed with a door and a mild foot shock (2 s, 0.3mA) was delivered to establish contextual fear memory. Twenty-four hours later retention memory was tested by placing the animal again in the lit box and latency to enter the dark compartment was timed, with 300 s as a cut-off.

### qPCR analysis

For the comparison of gene expression, we extracted brains from the CORT animals after testing and included age matched naive animals as baseline condition. After behavioural testing, RNA was isolated from the dorsal hippocampus with the Aurum kit (Biorad) according to the manufacturer’s instructions. RNA concentration was measured with the Nanodrop 1000 [ThermoFisher Scientific) and 300–1000 ng RNA was used to prepare cDNA with Superscript II (Invitrogen). qPCR was performed using the Roche LightCycler 480 system (Applied Biosystems). The best-performing housekeeping genes were determined by Genorm [71]. Results are given as relative expression (scaled to average) values normalized to the geometric mean of the housekeeping genes. Primers used for qPCR are depicted in Table 1. We selected the target genes based on their relevance for GC stimulation: neurogenesis and synaptic function (*Bdnf*, *TrkB*, *Fmr1*), sensitivity of cells to GC stimulation (*Fkbp5*), and dopaminergic modulation of cognitive processes in the hippocampus (*D1R*, *D5R*).

**Table 1:** Primer sequences qPCR.

Gene	Forward primer sequence	Reverse primer sequence
*Bdnf*	TTACCTGGATGCCGCAAACAT	TGACCCACTCGCTAATACTGTC
*Fkbp5*	TGAGGGCACCAGTAACAATGG	CAACATCCCTTTGTAGTGGACAT
*TrkB*	CTGGGGCTTATGCCTGCTG	AGGCTCAGTACACCAAATCCTA
*Drd1*	GTCTCCCAGATCGGGCATT	AGTCACTTTTCGGGGATGCT
*Drd5*	CTCGGCAACGTCCTAGTGTG	AATGCCACGAAGAGGTCTGAG
*Fmr1*	CAAGGCTTGGCAGGGTATGG	TCTCCAAACGCAACTGGTCT

### Statistical analysis

Sample size calculation: Using preliminary data on CORT treatment of C57BL/6 animals, we obtained a standard deviation of SD = 4464 (23h CA) and an effect size of d = 1.72. Assuming a type 1 error of 5% (α = 0.05) and the power of 80%, we calculated a sample size of n = 7/group. We used 2-way ANOVA, and for post-hoc contrast comparisons Sidak or Tukey corrections for multiple testing (GraphPad Prism® 7 for Windows). For SPSN, we performed 3-way ANOVA (Fisher’s least significant difference post hoc analysis) in Genstat. Animal numbers differed between baseline and CORT treatment, due to loss of animals as a consequence of higher fatal audiogenic seizure susceptibility of the FVB strain [48]. Data are expressed as means ±SEMs.

## Results

### Explorative and emotional behaviour

#### Cage activity

**Fig 2:**
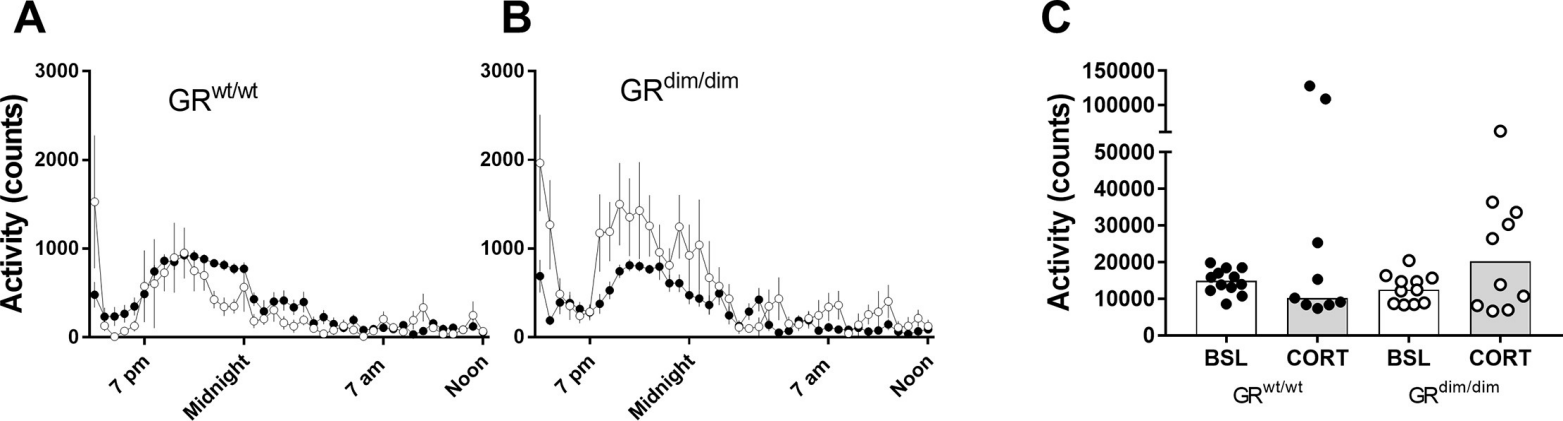
Overnight cage activity in GR^wt/wt^ and GR^dim/dim^ mice at baseline (BSL) and under chronic corticosterone (CORT) treatment. (A) In GR^wt/wt^ animals cage activity showed a typical activity pattern over 20h recording at baseline (black symbols, n = 12) or under chronic corticosterone (white symbols, n = 7). (B) GR^dim/dim^ animals show a similar activity pattern under baseline condition (black symbols, n = 11). Chronic corticosterone treatment (white symbols, n = 10) increased activity during the night period, but the contrast was not significant. Data are presented as means ±SEMs. (C) Total activity counts show the extreme increase in cage activity in 2 GR^wt/wt^ animals. Each data point reflects one animal, and bars indicate level of median. Baseline: n = 12 GR^wt/wt^ and n = 11 GR^dim/dim^ mice; CORT: n = 7 GR^wt/wt^ and n = 10 GR^dim/dim^ mice.

Under baseline conditions, we observed a typical activity pattern (low activity during the light phase and high activity during the night) and no differences between GR^wt/wt^ and GR^dim/dim^ mice [F(1,21) = 1.885, p = 0.1843], as visualized in the overnight activity pattern (30 min time bins, Fig 2A and 2B, black symbols) as well as the total counts (Fig 2C). Under chronic corticosterone schedule, most GR^wt/wt^ animals (7 of 9) showed no effect of corticosterone, however, 2 GR^wt/wt^ animals displayed an extreme increase in activity (> 100 000 counts in 23h, Fig 1C). While in GR^dim/dim^ activity was increased in 5 out of 10 animals (Fig 2B and 2C). Two-way ANOVA (excluding the 2 extreme GR^wt/wt^ animals) indicated no genotype [F(1,34) = 1.909, p = 0.176) and no treatment effect [F(1,34) = 2.29, p = 0.139) (Fig 2C).

#### Open field

**Fig 3:**
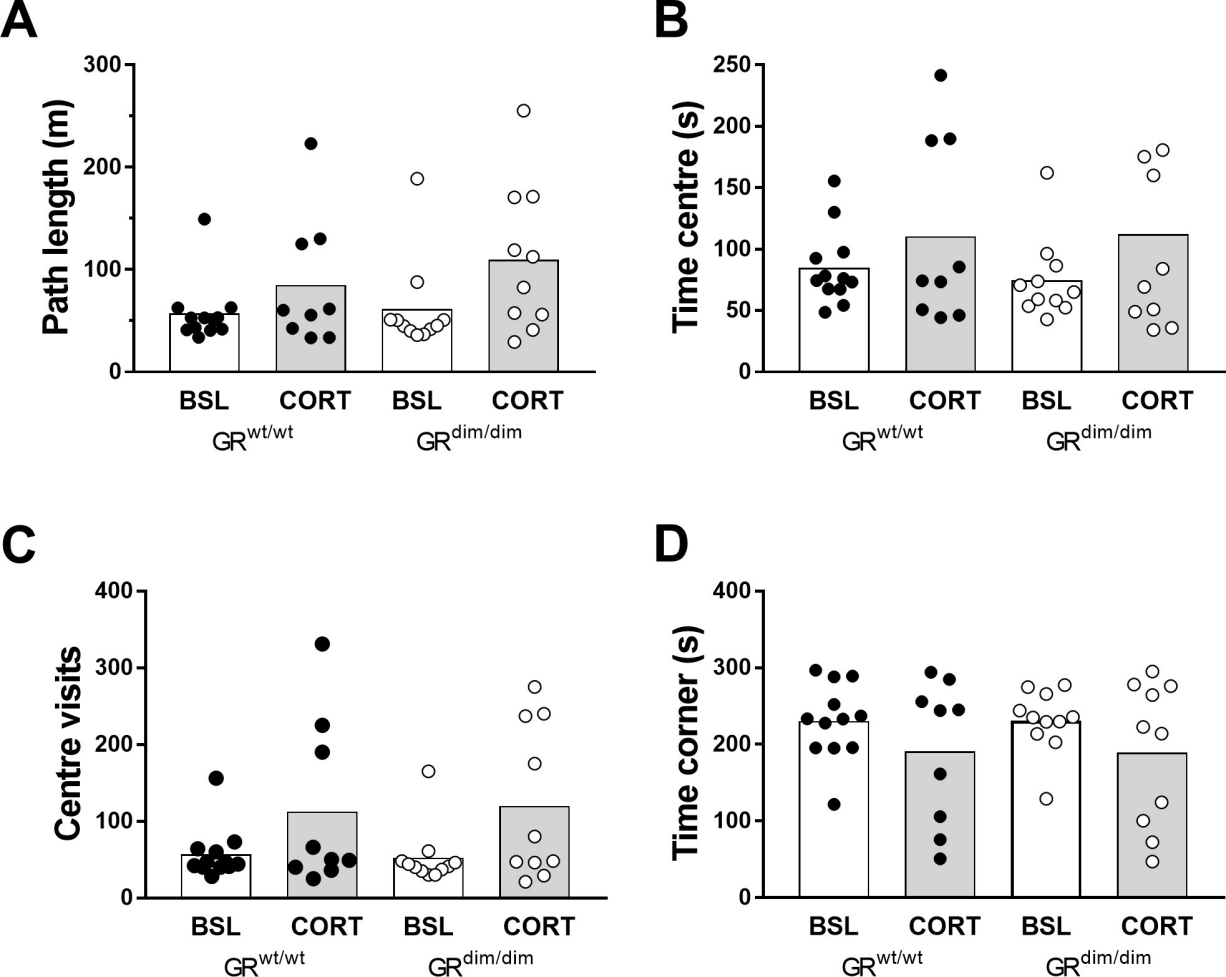
Explorative behaviour in the open field in GR^wt/wt^ and GR^dim/dim^ mice at baseline (BSL) and under chronic corticosterone (CORT) treatment. When placed in an open field, overall exploration was similar in both genotypes expressed as path length (A), as time spent in the centre (B), and number of centre visits (C), and as time spent in the 4 corners (D). Data are expressed as means ± SEMs. Baseline: n = 12 GR^wt/wt^ and n = 11 GR^dim/dim^ mice; CORT: n = 9 GR^wt/wt^ and n = 10 GR^dim/dim^ mice.

Exploration in the open field was assessed by measuring path length (Fig 3A). In addition, conflict resolution related readouts, such as centre time (Fig 3B), centre visits (Fig 3C) and thigmotaxis (wall hugging or corner time) (Fig 3D) were extracted from video-based tracking. We observed no genotype effect in path length [F(1,38) = 0.7552, p = 0.39], and only an effect of treatment [F(1,380 = 5.319, p = 0.026], without interaction. (Fig 3A). Furthermore, time in centre was similar in both genotypes [F91,38) = 0.053, p = 0.81] and treatment had no effect [f(1,38) = 3.083, p = 0.087]. Number of visits to the centre were not different between genotypes [F(1,38) = 0.004, p = 0.949], while factor treatment was [F(1.38) = 6.99, p = 0.0118]. Similarly, no genotype effect was observed for time spent in the corners [F(1,38) = 0.0006, p = 0.980], nor for treatment [F(1,38) = 3.313, p = 0.766]. However, under corticosterone treatment, we also observed a dramatic change in activity in some animals (in 3 GR^wt/wt^ and in 4 GR^dim/dim^ mice), in particular an increase in body rotations (S1 Fig). Overall, we observed similar explorative patterns between the two genotypes at baseline and under CORT treatment.

#### Elevated plus maze

We performed the elevated plus maze only at baseline, since under corticosterone, the animals were too active to stay on the setup and made recording impossible. At baseline, GR^dim/dim^ mice crossed into open arms significantly more often when compared to GR^wt/wt^ mice [t(21) = 2.26, p = 0.0346] (S2 Fig). In conjunction with similar general activity [total arm entries, t(21) = 0.93, p = 0.3629], this suggests that GR^dim/dim^ are less anxious than GR^wt/wt^ (S2 Fig).

### Social behaviour and cognition

#### Sociability and Preference of Social Novelty (SPSN)

**Fig 4:**
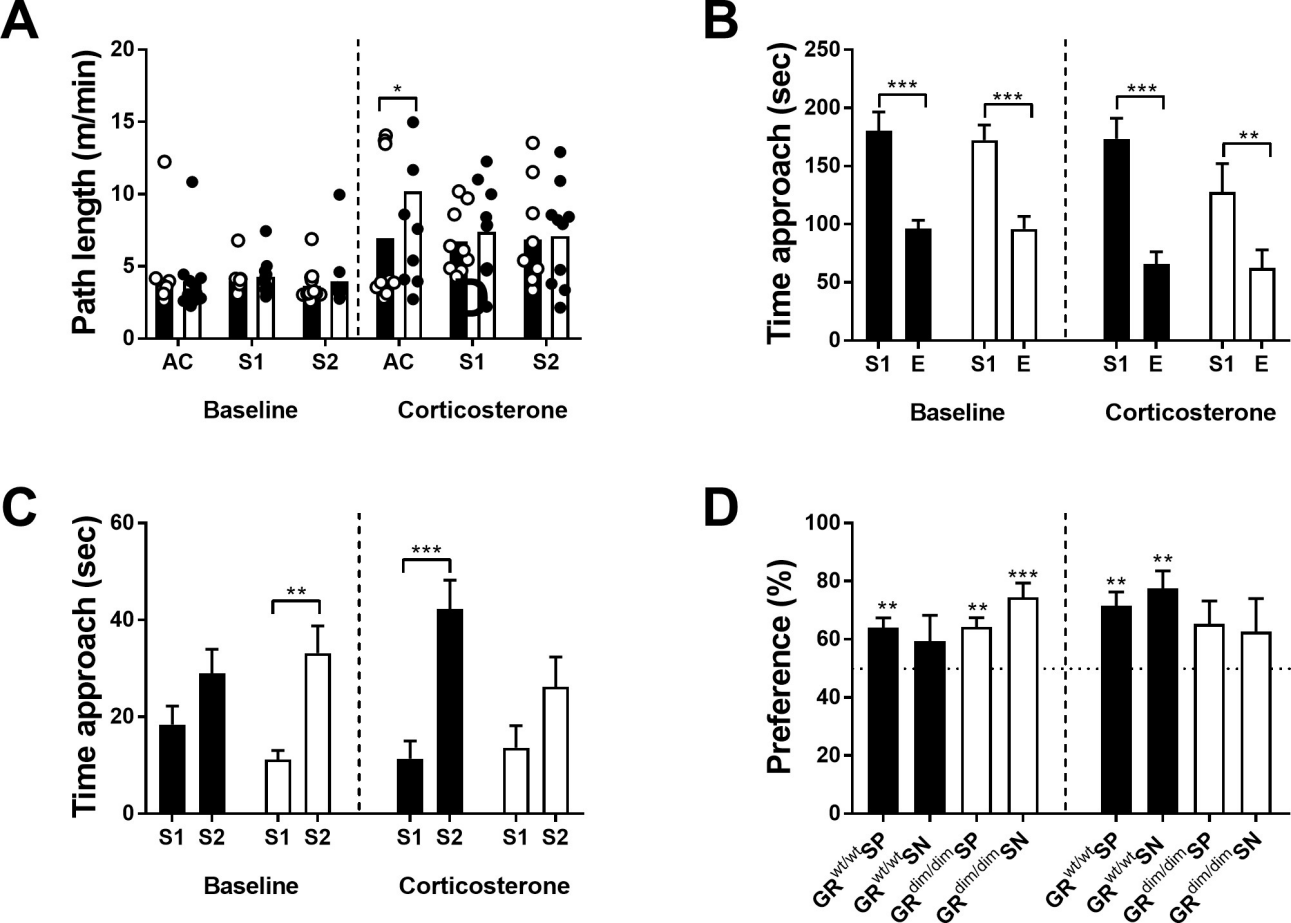
Sociability (SP) and preference for social novelty (SN) in GR^wt/wt^ (black bars) and GR^dim/dim^ mice (white bars) under baseline and under chronic corticosterone treatment. Under baseline conditions, no difference in path length (m/min) was observed between the genotypes.CORT treatment increased path length (m/min) in both genotype similarly in comparison to baseline (A). When presented with a stranger mouse (S1), under baseline and CORT conditions, both genotypes showed more approach behaviour towards S1 than to an empty (E) cage (B). When presented with a second stranger (S2), under baseline conditions GR^dim/dim^ mice show a preference towards S2, while under CORT treatment, GR^dim/dim^ mice do not display preference towards S2 anymore (C). Calculation of individual preference scores indicate that under baseline conditions, both genotypes show normal social preference (SP: S1-E), however, only GR^dim/dim^ mice show preference for social novelty (SN: S1-S2). Under CORT, GR^wt/wt^ show preference during SN and SP, while GR^dim/dim^ mice show no preference in SP and SN (D). Data are shown as means ± SEMs. (B-D) * p < 0.05, **p < 0.01 ** p < 0.01, ***p < 0.001, (one sample t-test to chance level at 50%). Baseline: n = 12 GR^wt/wt^ and n = 11 GR^dim/dim^ mice; CORT: n = 9 GR^wt/wt^ and n = 10 GR^dim/dim^ mice.

Path lengths (m/min) during each of the stages were compared using 3-way ANOVA for factor stage (AC, S1, S2), genotype (GR^wt/wt^ and GR^dim/dim^) and treatment (baseline or corticosterone) (Fig 4A). We only found an effect for treatment [F(1, 114) = 37.17, p < 0.001].

During the sociability phase (SP), mice are presented either a stranger mouse (S1) or an empty (E) cup and time sniffing (distance < 2cm from cup) was scored. Three-way ANOVA for factor side, genotype and treatment indicated a significant effect for side [F(1,76) = 61, p < 0.001), and for treatment [F(1,61) = 7.45, p = 0008], but no effect for genotype [F(1,76) = 1.97, p = 0.164]. (Fig 4B). Post hoc comparisons (Fisher’s least significant difference (LSD) test), indicated both genotypes investigated S1 longer than the empty cage, in baseline conditions and with CORT treatment (GR^wt/wt^: p < 0.001 in baseline and CORT, GR^dim/dim^: p < 0.001 and p < 0.01 in baseline and CORT respectively).

In the last stage, a second stranger is added to the empty wire cage and social memory can be assessed. Usually, mice will show higher preference to the novel stranger (S2)., Three-way ANOVA for factor side, genotype and treatment, indicated a significant effect for side [F(1,76) = 30.25, p < 0.001], but not for genotype [F(1,76) = 1.39, p = 0.243] and treatment [F(1,76) = 0.01, p = 0.908]. There was also a significant interaction between the three factors [F(1,76) = 4.88, p = 0.03]. (Fig 4C). Post hoc analysis with LSD test revealed that, under baseline conditions, GR^dim/dim^ mice showed more interest in S2 (p < 0.01), while GR^wt/wt^ animals did not (p > 0.05). When treated with CORT, GR^wt/wt^ animals showed an increase interest in S2 (p < 0.001), while GR^dim/dim^ mice did not(p > 0.05), indicating that CORT affected recognition for the novel stranger (S2).

To account for inter-individual differences in time, we calculated the preference score for S1 (in SP) and for S2 (in SN) under baseline and under CORT conditions (Fig 4D). Under baseline conditions, GR^dim/dim^ displayed a significant contrast to chance level (50%) (one sample t-test to 50%: GR^dim/dim^ SP: [t(10) = 4.52, p = 0.001], SN: [t(10) = 4.98, p = 0.0006]), while GR^wt/wt^ only in SP [t(11) = 4.166, p = 0.0016], but not during SN [t(11) = 1.06], indicating reduced social memory under baseline conditions. Under CORT treatment, GR^wt/wt^ displayed a significant preference above chance level (SP:[t(8) = 4.599, p = 0.0018]; SN: [t(8) = 4.556, p = 0.0019], while in GR^dim/dim^ the preferences were not different from chance level (SP: [t(8) = 1.941]; SP: t(8) = 1.109] indicating that in GR^dim/dim^ corticosterone treatment affected both preference towards S1 and S2.

#### Spontaneous alternation in Y maz

**Fig 5:**
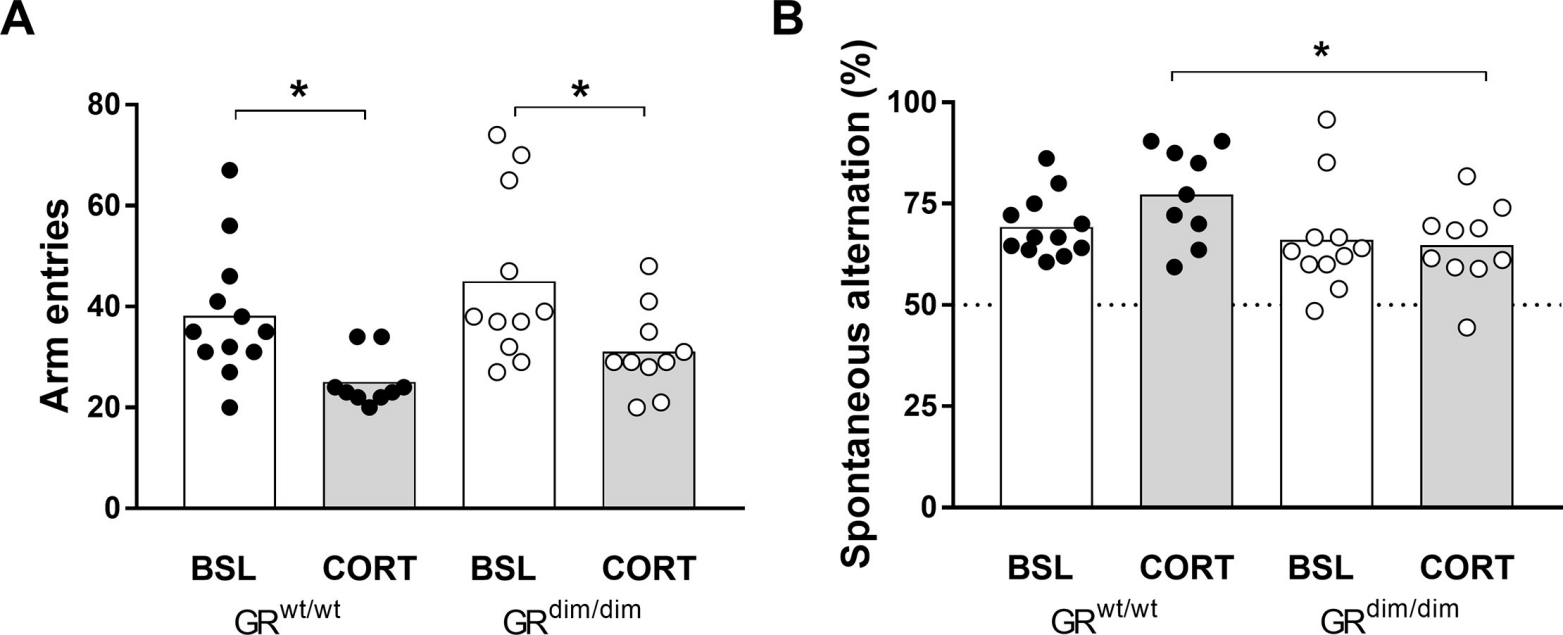
Spontaneous alternation Y-maze. CORT reduced the number of arm visits compared to baseline in both genotype (A). In contrast, spontaneous alternations were higher in GR^wt/wt^ mice (B). Data are shown as mean ± SEM. *p < 0.05. Baseline: n = 12 GR^wt/wt^ and n = 11 GR^dim/dim^ mice; CORT: n = 9 GR^wt/wt^ and n = 10 GR^dim/dim^ mice.

Working memory was assessed in the Y-maze. 2-way ANOVA for arm visits indicated an effect for treatment [F(1,38) = 13.04, p = 0.0009], without an effect for genotype [F(1,38) = 2.895, p = 0.097], indicating that under CORT number of arm visits were reduced (Fig 5A). When we compared spontaneous alternations (SA), 2-way ANOVA indicated that there was no effect for factor treatment [F(1,38) = 1.013, p = 0.3216], but a significant effect for genotype [F(1,380 = 5.467, p = 0.0247] (Fig 5B). Working memory in the Y-maze is based on the notion that an animal remembers the two arms it visited last and will choose the most novel arm to enter. This process implies that when the animal arrives at the decision point where all 3 arms come together, it might briefly pause and then make the decision which arm to visit. We observed that the SA were similar in GR^wt/wt^ at baseline and under CORT. However, the significantly reduced number of arm visits could be an indication, that the decision might be more difficult and might require more time. Indeed the amount of SA are slightly higher under CORT in GR^wt/wt^. In contrast, in GR^dim/dim^ mice, number of visits also decreased under CORT, however, the amount of SA was the same as under baseline conditions. In addition, the SA under CORT was significantly lower than in GR^wt/wt^ mice, which could point to a reduced hippocampal function in GR^dim/dim^ mice when challenged with CORT.

#### Passive avoidance

**Fig 6:**
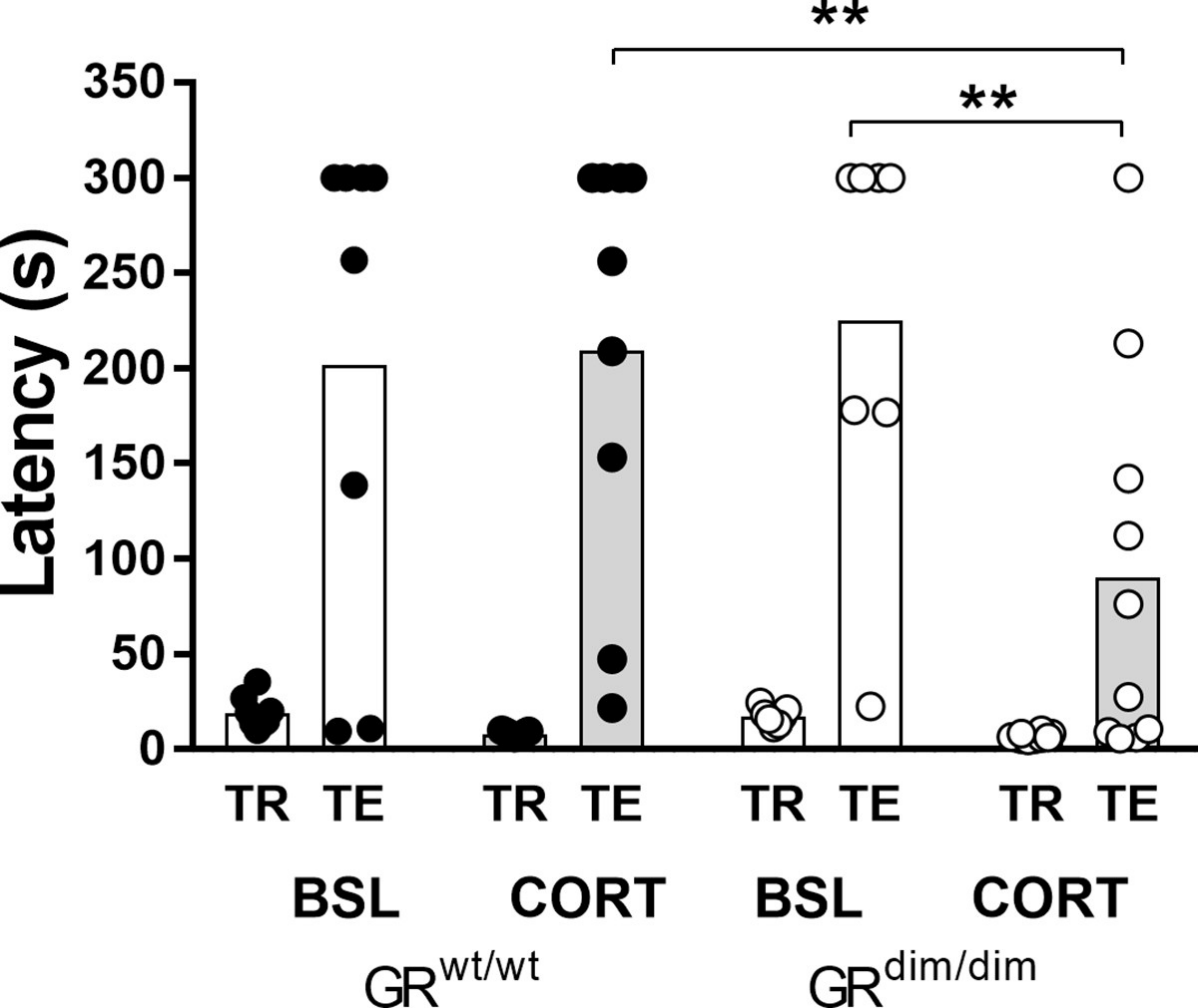
Passive avoidance. During training (TR), the animal received a mild foot shock upon entering the dark compartment. 24h later during testing (TE), entry to the dark compartment is avoided when contextual memory is intact. No differences were observed at baseline conditions (BSL). After CORT treatment, the latency in GR^dim/dim^ mice was significantly lower when compared to BSL, as well as to GR^wt/wt^ mice. Data are presented as mean +/- SEM. ** p < 0.01 (2-way ANOVA, post hoc with Sidak correction). Baseline: n = 12 GR^wt/wt^ and n = 11 GR^dim/dim^ mice; CORT: n = 9 GR^wt/wt^ and n = 10 GR^dim/dim^ mice.

Contextual fear memory was assessed in a passive avoidance test. Latency to enter the dark compartment was measured during training (TR) and test (TE) phase. Under baseline conditions, we observed similar fear memory retention during the test phase. Animals avoided entering the box that had been associated with a mild foot shock (Fig 6). Under CORT schedule, GR^wt/wt^ mice still avoided to enter, while GR^dim/dim^ mice entered the dark box on average after 100s, indicating that contextual fear memory in GR^dim/dim^ mice was significantly impaired after chronic corticosterone treatment. Two-way ANOVA indicated a significant effect for phase [F(1,17) = 33.84, p < 0.0001) and a group effect [F(1,17) = 6.084, p = 0.025) with a significant interaction [F(1,17) = 5.8, p = 0.0277). Post-hoc analysis using Sidak’s correction indicated that under CORT the latency to enter for GR^dim/dim^ was significantly different from baseline [q(60) = 4.864, p = 0.0057], as well as from GR^wt/wt^ mice [t(60) = 4.613, p = 0.0096], and therefore, under chronic corticosterone treatment, GR^dim/dim^ mice have impaired contextual fear memory retention.

#### Conditioned place preference (CPP) test

**Fig 7:**
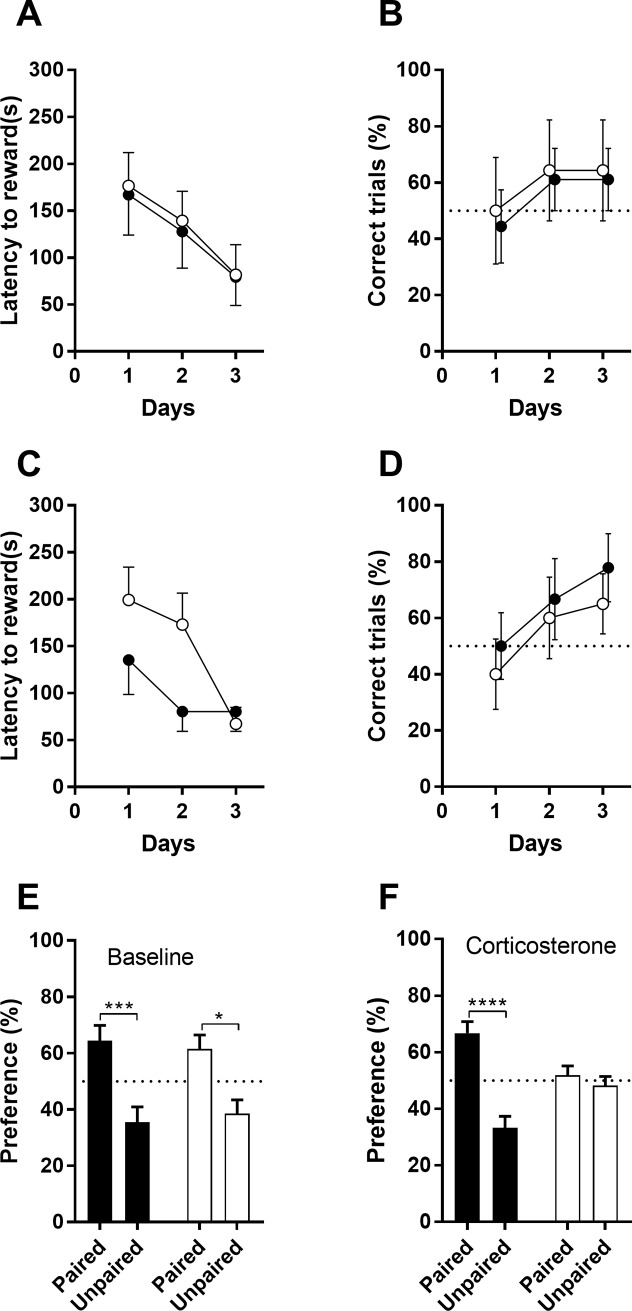
Conditioned place preference in GR^wt/wt^ (black symbols) and GR^dim/dim^ mice (white symbols). Animals were conditioned to locate a food reward in a specific context for 3 days (6 trials). Latency to find the reward (A, C) reduced over trials, and number of correct trials (B, D) increased over training trials. During training, no effect of genotype was found under baseline conditions (A, B) and not under CORT treatment (C, D). During testing, both genotype show clear preference for the correct context under baseline conditions (E), while under CORT treatment, GR^dim/dim^ did not display a context preference (F). Data are shown as mean ± SEM. * p < 0.05. *** p < 0.001, **** P < 0.0001. Baseline: n = 9 GR^wt/wt^ and n = 7 GR^dim/dim^ mice; CORT: n = 9 GR^wt/wt^ and n = 10 GR^dim/dim^ mice.

Incentive salience and contextual memory can be measured by the amount of time the mice spend in the food-paired compartment during a probe trial. We trained animals for a limited number of trials (6 trials) to associate a food reward with a certain context. A reduction of time to eat the reward, as well as increase in correct choices indicated learning. Under baseline conditions, both GR^wt/wt^ and GR^dim/dim^ mice were able to learn to locate the food reward in the correct context (Fig 6A, 2-way RM ANOVA day [F(2,28) = 6.305; p = 0.0055, but no effect for genotype [F(1,14) = 0.033)]). While latency decreased, the number of correct trials displayed a trend to be above chance level (correct trials = 50%) (Fig 7B). During the probe trial, all animals displayed a significant preference to the correct context that was associated with a reward [F(1,28) = 23.57, p < 0.0001] (Fig 7E), without effect for genotype [F(1,28) = 0, p > 0.99].

Under corticosterone treatment, all animals showed a decrease in latency to collect the food over days [F(2,34) = 8.69; p = 0.0009], but no genotype effect [F(1,17) = 2.344] (Fig 7C), and no significant interaction [F(2,34) = 2.948, p = 0.066]. Post-hoc comparison for days, signalled that GR^dim/dim^ animals improved latency to find the food reward on the third day significantly over the first day [t(51) = 3.335, p = 0.0048], while in GR^wt/wt^ animals the time to find the food was similarly low over the 3 days. Number of errors displayed a trend to be above chance level (Fig 7D). Under corticosterone treatment, 2-way ANOVA indicated a significant effect of context [F(1,34) = 25.45, p < 0.0001], as well as significant interaction [F(1,34) = 16.24, p = 0.0003]. Posthoc analysis revealed that only GR^wt/wt^ [t(34) = 6.254, p < 0.0001], but not GR^dim/dim^ mice [t(34) = 0.737, p = 0.71], showed a significant preference for the paired context (Fig 7F).

### Gene expression analysis in dorsal hippocampus

**Fig 8:**
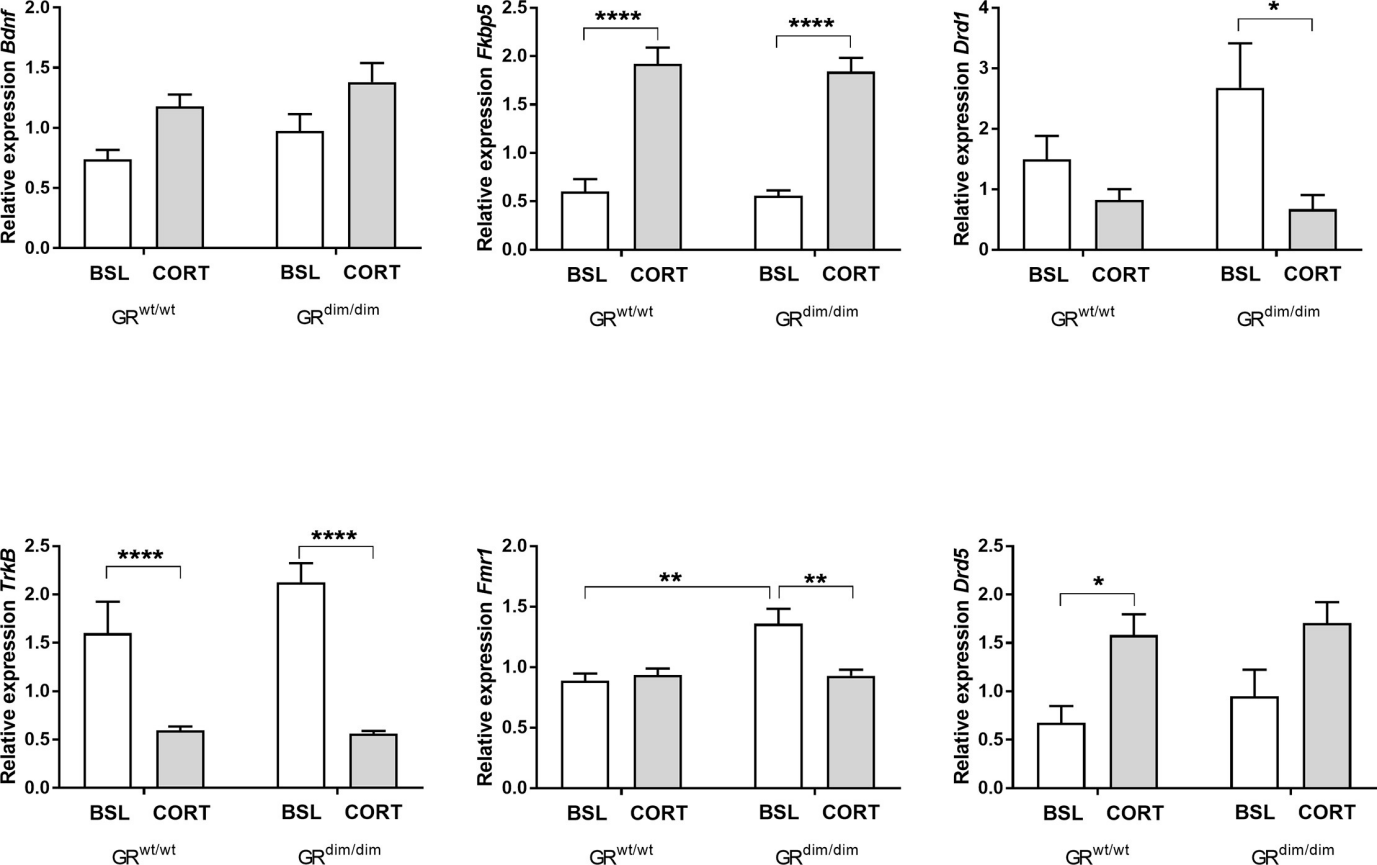
Gene expression level changes in the dorsal hippocampus of genes associated with increases of corticosterone (*BDNF*, *TRKB*, *Fkbp5*, and *Fmr1*) and stress and cognition (*Drd1*, *Drd5*). Tissue was extracted from animals after behavioral testing (n = 6) and compared to experimental naïve animals as baseline controls (n = 6). Data are presented as mean +/- SEM as relative expression, normalized to housekeeping genes and scaled to the average across all unknown samples per target, * p < 0.05; ** p < 0.01; *** p < 0.0001 (2-way ANOVA and post-hoc comparison with Sidak’s correction for multiple testing).

We compared hippocampal expression of a selection of genes associated with neurogenesis and synaptic pruning (*Bdnf*, and its receptor *TrkB*, and *Fmr1)*, cellular sensitivity to GC stimulation *(Fkbp5)*, and modulation of cognitive processes as well as to the salience network (*Drd1*, *Drd5*). The gene expression levels are shown as relative expression, scaled to average and normalized to housekeeping genes. We compared all expression levels using a 2-way ANOVA on the log-transformed data with genotype and treatment as factors. Overall, we found that CORT treatment significantly increased expression levels of *Bdnf*, *Fkbp5* and *Drd5*, and decreased expression of *TrkB* and *Drd1* (see Table 2, and Fig 8). For these genes, the contrast between the genotypes was not significant, and we also found no interaction. *Fmr1* comparison indicated a significant genotype effect, treatment effect as well as a significant interaction. Post-hoc contrast analysis (Sidak’s multiple testing corrections) indicated that *Fmr1* was significantly higher in GR^dim/dim^ at baseline [t(20) = 4.232, p = 0.0025] and that CORT treatment returned *Fmr1* expression back to GR^wt/wt^ levels [t(20) = 3.734, p = 0.0078; GR^dim/dim^ BSL versus CORT; t(20) = 0.074, p = > 0.99, GR^wt/wt^ versus GR ^dim/dim^ under CORT].

**Table 2:** Statistical analysis of gene expression levels using 2-Way ANOVA on the log transformed results.

Gene	*genotype*	*treatment*	*genotype x treatment*
	DFn, DFd = 1,10		
*Bdnf*	F = 2.371, p = 0.139	F = 11.22, p = 0.003	F = 0.191, p = 0.666
*TrkB*	F = 1.832, p = 0.191	F = 100.7, p < 0.0001	F = 3.203, p = 0.0887
*Fkbp5*	F = 0.010, p = 0.921	F = 88.84, p < 0.0001	F = 0.042, p = 0.839
*Fmr1*	F = 8.645, p = 0.0081	F = 4.990, p = 0.037	F = 9.269, p = 0.0064
*Drd1*	F = 0.153, p = 0.699	F = 10.60, p = 0.004	F = 2.278, p = 0.146
*Drd5*	F = 0.689, p = 0.416	F = 13.27, p = 0.0016	F = 0.210, p = 0.651

## Discussion

In this study we analysed the behavioural phenotype of female FVB/NJ mice harbouring a point mutation in the glucocorticoid receptor (GR), known as GR^dim/dim^, under baseline and under chronically elevated corticosterone levels. In particular, we compared behaviour spanning general activity, emotional behaviour and cognitive performance under these two conditions. In addition, we analysed expression levels of selected genes that have been shown to be differentially regulated by (i) corticosterone [72–74] and stress [75], (ii) in major depressive disorders in the hippocampus [29], and (iii) play an important role in cognitive processes especially in learning impairments reported in preclinical models and clinical population of depression [76–80]. Tests relying on visual cues were not included since FVB/NJ mice have been shown to be vision-impaired [81].

Previous behavioural reports on animals with targeted disruption of glucocorticoid receptor in a C57BL/6J background have demonstrated impaired learning and reference memory in a short Morris water maze protocol [82, 83], paired with increased explorative behaviour in an open field and /or in a dark/light box was. In addition, in these GR^dim/dim^ animals (C57BL/6J background), the circulating corticosterone levels were elevated, both at trough and peak levels, as well after acute stress evoked by forced swimming [83]. In general, C57BL/6J animals have lower baseline corticosterone levels than FVB/NJ animals (e.g. at baseline 27.5 ng/ml in comparison to 41.5 ng/ml as reported by [84]), which reflects differences in behavioural despair anxiety-related exploration in the open field [84]. However, despite increased baseline levels due to genetic background, GR^wt/wt^ animals in a FVB/NJ background displayed sensitivity to the protective effects of dexamethasone in a TNF-induced lethal inflammation model [85], which would indicate that the HPA-axis feedback regulation is not desensitized [85]. Despite the reported cognitive differences in the C57BL/6J background, we observed no genotype-specific differences in cognitive nor in emotional behaviour in this GR^dim/dim^ model (with the exception of a small contrast in open arm visits in the elevated plus maze). Because we did not observe any difference between GR^dim/dim^ mice and GR^wt/wt^ mice under baseline conditions, both groups were subjected chronically (4 weeks) to low doses of corticosterone (36,5 mg/l, CORT) in drinking water. This specific schedule has been shown to robustly induce anxiety and depression-like behaviour [51, 52, 86], including time spent in the centre of the open field, increased immobility in the forced swim test and suppressed novelty feeding, as well as impaired learning and memory [51], and reduced cell proliferation in the dendate gyrus of the adult hippocampus [52, 87]. To investigate the effect of chronic elevated corticosterone levels, we first treated the animals for 4 weeks and repeated behavioural testing while CORT was maintained at a steady state to avoid acute rebound effects. Corticosterone induced some genotype unspecific effects, such as hyperactivity in CA, OF, SPSN and EPM, at least in a subset of animals in both groups, which indicates that CORT effects in FVB/NJ background might be due to a general reduction in CNS inhibition. Indeed, FVB/NJ mice have been reported to have increased occurrence of audiogenic seizures and less sensitive to GABA_A_ ligands [48], both indicative of increased neuronal excitability in the FVB/NJ strain. This increase in activity was observed in both genotypes especially when placed in a novel environment, such as the OF, SPSN and in CA. Notably, while GR^wt/wt^ mice show initially high activity in the overnight cage activity, after a few hours activity reached baseline levels during the night. Overall, this indicates that this particular strain (FVB/NJ) is more sensitive to low levels of corticosterone by displaying increases in novelty-induced locomotion.

In contrast to the genotype unspecific changes in activity, we did observe a genotype-specific effect of CORT on cognitive behaviour in 4 different tests. In particular, we observed reduced social recognition, reduced spontaneous alternation in the Y-maze, reduced contextual fear memory in a passive avoidance test, and reduced contextual place preference in GR^dim/dim^ mice under CORT treatment. These cognitive impairments are very likely independent of the changes in activity levels, because we observe similar increase in locomotion in GR^wt/wt^ mice without cognitive impairment. Despite increased activity, GR^wt/wt^ mice show perfect avoidance by not entering the conditioned context. In tests with higher cognitive load such as working memory in the Y-maze, number of arm visits were reduced in GR^wt/wt^ mice under CORT, while the number of alternations were above chance level. Glucocorticoids (GC), commonly prescribed over extended periods to suppress inflammation and immune responses, have been reported to cause a variety of behavioural changes, including cognitive impairment [88]. Furthermore, numerous studies suggest a strong association between elevated GC hormone levels and hippocampal damage [89]. The hippocampus is a major hub for cognitive processes in the mammalian brain, especially for episodic, spatial and contextual information, but it is also exceptionally well positioned to detect and respond to stress because of its rich concentration of GC receptors [90, 91]. Long term exposure to stress or GC results in numerous changes in the hippocampal structure including neurochemistry, excitability, neurogenesis, neuronal morphology and even cell death [92].

In our model, we observe an effect of CORT on hippocampus-dependent cognition only in GR^dim/dim^ mice. There are several possible explanations for this observation. Firstly, we chose a concentration of CORT that was reported to induce behavioural and cognitive changes in mice and rats, but is considered a rather low dose [55], reflecting a 3-fold elevation of baseline corticosterone in C57BL/6J animals. Baseline levels in FVB have been reported to be higher [84]. Therefore it is possible that the amount we provided in the drinking water had less of an effect in GR^wt/wt^ mice in this genetic background. In addition, stress sensitivity has been shown to be different between specific mouse strains [93]. For example, Ibarguen-Vargas et al reported a differential effect of chronic stress on novelty feeding in C57BL/6 and FVB [93]. In C57Bl/6, stress increased the reluctance to approach food in a novel environment, but had no effect on novelty feeding in FVB [93]. Restraining stress induces anxiety-and depression like behaviour in C57Bl/6, but not in FVB/NJ [94]. Secondly, chronic stress manipulations or pharmacologically elevating corticosterone levels have been shown to impair cognitive flexibility (e.g. working memory [95], spatial reference memory [96], and contextual fear learning (e.g. passive avoidance [97, 98]) in a variety of rodent models, as well as in humans [99–101]. However, depending on the protocols, corticosterone treated animals perform similar to controls [95, 102–105]. So it is possible that in other cognitive protocols, e.g. in the hidden platform version of the Morris water maze, GR^wt/wt^ mice might have performed worse under chronic corticosterone. However, we chose not to perform cognitive tests that rely mostly on visual acuity with this strain, since FVB have been reported to develop retinal degeneration [106].

Oitzl et al reported impaired spatial memory for GR^dim/dim^ mice in the Morris water maze under basal conditions [83]. They argued that the observed cognitive deficit was based on the lack of DNA binding-dependent transcriptional regulation by GR, and not to the elevated levels of corticosterone at baseline and under stress [83]. Indeed, it is worth noting that exposure to water triggered a higher and much longer release of corticosterone in the GR^dim/dim^ mice. We did not observe cognitive deficits in GR^dim/dim^ mice under baseline conditions in the CPP, PA or Y-maze. This discrepancy could be in the test protocol that poses a different demand on cognitive function. Spatial learning in the Morris water maze, as well as place preference, passive avoidance, and spontaneous alternation all rely on hippocampal processes, but they differ in their cognitive load [107]. So it is possible that our protocols were easier to learn and therefore no genotype effect was evident. In addition, we performed an extensive behavioural battery, employing a variety of behavioural protocols in a well-defined order. However, test order, as well as test history has been shown to affect mouse behaviour and even mask cognitive deficits [108, 109].

The molecular picture of GC action in the brain is far from complete. However, it is clear that GCs exert direct transcriptional effects on many different components of the neurotransmission cascade. These components include patterns of gene expression underlying signal transduction, neuronal structure, vesicle recycling, neurotransmitter catabolism, motor activity, cell adhesion, neuronal outgrowth and survival and energy metabolism [110]. Because, in general, GR^dim/dim^ phenotypes are associated with a lack in transcriptional control of GR dimers, and because the observed phenotypes are hippocampus controlled, we investigated gene expression of different key genes in the dorsal hippocampus. We focussed on genes that have been shown to be important in cognitive processes and are associated with stress and MDD, such as *Bdnf* and its receptor *TrkB*, D1-like receptors (*Drd1* and *Drd5*) and *Fmr1*. In addition, we also included *Fkbp5*, which regulates GR sensitivity to GC, and overexpression of *Fkbp5* has been associated with increased risk of anxiety and MDD in patients [111]. We found that CORT increased expression levels of *Bdnf*, *Fkbp5 and Drd5*, while *Drd1* and *TrkB* were downregulated. Both the upregulation of *Fkbp5* and downregulation of *TrkB* would be expected as a consequence of chronic GC levels. Upregulation of *Fkbp5* is induced by increases in GC levels via intronic hormone response elements acting as a short negative feed-back loop [111]. Similarly, down regulation of BDNF/TrkB pathway is a hallmark of stress-induced changes in mouse and in MDD [112]. Changes in *Fkbp5* and *Bdnf/TrkB* expression support the behavioural observations under chronic CORT administration. Interestingly, it seems the CORT administration affected the expression of *Drd1* and *Drd5* differentially. Both receptors are located in the hippocampus and have been attributed to learning and memory processes. However, due to lack of subtype specific ligands, there is only limited information regarding the subtype specific influences on cognitive processes. Furthermore, *Drd1* is much more abundant than *Drd5*, which made interpretation of behavioural phenotype of for example *Drd5* specific knockout difficult. However, there is growing evidence, that *Drd5* as well as *Drd1* are involved in cognitive processes by either modulating NMDA receptors or by releasing acetylcholine [113–115]. We observed a difference in CORT regulation of the *Fmr1* gene expression between GR^wt/wt^ and GR^dim/dim^ mice. The data suggest that the phenotypic problem in containing a good memory after chronic stress in GR^dim/dim^ mice is not reflected in the lack of induction of repression of these genes in the hippocampus, and it may be that other genes, not tested here or as yet not recognized, are regulated by GR dimers in this specific control.

## Supporting information

S1 Fig Open field. Two examples of increased rotational behaviour after CORT treatment. The two panels on the left show respective tracks for the first 20s of the open field experiment in two mice (A and B) under baseline conditions. Under CORT, the behaviour changes dramatically to fast running in circles in the same mice.
(TIF)

S2 Fig Elevated plus maze. GR^dim/dim^ mice crossed into open arms significantly more, relative to the closed arm, when compared to controls (B). In conjunction with similar general activity [total crosses) (A), indicates that GR^dim/dim^ mice are less anxious than WT controls. Data are presented as mean +/- SEM. * denotes p < 0.05 between genotypes.
(TIF)

## References

[1] Smith SM, Vale WW. The role of the hypothalamic-pituitary-adrenal axis in neuroendocrine responses to stress. Dialogues Clin Neurosci. 2006;8(4):383–95. 17290797 10.31887/DCNS.2006.8.4/ssmith PMC3181830

[2] Roozendaal B, McEwen BS, Chattarji S. Stress, memory and the amygdala. Nat Rev Neurosci. 2009;10(6):423–33. 10.1038/nrn2651 . 19469026

[3] Joels M, Karst H, Sarabdjitsingh RA. The stressed brain of humans and rodents. Acta Physiol (Oxf). 2018;223(2):e13066 10.1111/apha.13066 29575542 PMC5969253

[4] Herman JP, McKlveen JM, Ghosal S, Kopp B, Wulsin A, Makinson R, et al Regulation of the Hypothalamic-Pituitary-Adrenocortical Stress Response. Compr Physiol. 2016;6(2):603–21. 10.1002/cphy.c150015 27065163 PMC4867107

[5] Saaltink DJ, Vreugdenhil E. Stress, glucocorticoid receptors, and adult neurogenesis: a balance between excitation and inhibition? Cell Mol Life Sci. 2014;71(13):2499–515. 10.1007/s00018-014-1568-5 24522255 PMC4055840

[6] Duman RS, Aghajanian GK. Synaptic dysfunction in depression: potential therapeutic targets. Science. 2012;338(6103):68–72. 10.1126/science.1222939 23042884 PMC4424898

[7] Beato M, Klug J. Steroid hormone receptors: an update. Hum Reprod Update. 2000;6(3):225–36. 10.1093/humupd/6.3.225 . 10874567

[8] Boumpas DT, Chrousos GP, Wilder RL, Cupps TR, Balow JE. Glucocorticoid therapy for immune-mediated diseases: basic and clinical correlates. Ann Intern Med. 1993;119(12):1198–208. 10.7326/0003-4819-119-12-199312150-00007 . 8239251

[9] Cole TJ, Blendy JA, Monaghan AP, Schmid W, Aguzzi A, Schutz G. Molecular genetic analysis of glucocorticoid signaling during mouse development. Steroids. 1995;60(1):93–6. 10.1016/0039-128x(94)00009-2 . 7792824

[10] Marques AH, Silverman MN, Sternberg EM. Glucocorticoid dysregulations and their clinical correlates. From receptors to therapeutics. Ann N Y Acad Sci. 2009;1179:1–18. 10.1111/j.1749-6632.2009.04987.x 19906229 PMC2933142

[11] Kanatsou S, Joels M, Krugers H. Brain Mineralocorticoid Receptors and Resilience to Stress. Vitam Horm. 2019;109:341–59. 10.1016/bs.vh.2018.11.001 . 30678862

[12] de Kloet ER, Joels M, Holsboer F. Stress and the brain: from adaptation to disease. Nat Rev Neurosci. 2005;6(6):463–75. 10.1038/nrn1683 . 15891777

[13] Joels M, Krugers HJ, Lucassen PJ, Karst H. Corticosteroid effects on cellular physiology of limbic cells. Brain Res. 2009;1293:91–100. 10.1016/j.brainres.2009.03.036 WOS:000270865600010. 19332034

[14] Groeneweg FL, Karst H, de Kloet ER, Joels M. Mineralocorticoid and glucocorticoid receptors at the neuronal membrane, regulators of nongenomic corticosteroid signalling. Mol Cell Endocrinol. 2012;350(2):299–309. 10.1016/j.mce.2011.06.020 . 21736918

[15] Chauveau F, Tronche C, Pierard C, Liscia P, Drouet I, Coutan M, et al Rapid stress-induced corticosterone rise in the hippocampus reverses serial memory retrieval pattern. Hippocampus. 2010;20(1):196–207. 10.1002/hipo.20605 . 19360856

[16] Dorey R, Pierard C, Shinkaruk S, Tronche C, Chauveau F, Baudonnat M, et al Membrane mineralocorticoid but not glucocorticoid receptors of the dorsal hippocampus mediate the rapid effects of corticosterone on memory retrieval. Neuropsychopharmacology. 2011;36(13):2639–49. 10.1038/npp.2011.152 21814189 PMC3230488

[17] Keller J, Gomez R, Williams G, Lembke A, Lazzeroni L, Murphy GM Jr., et al HPA axis in major depression: cortisol, clinical symptomatology and genetic variation predict cognition. Mol Psychiatry. 2017;22(4):527–36. 10.1038/mp.2016.120 27528460 PMC5313380

[18] Claes S. Glucocorticoid receptor polymorphisms in major depression. Ann N Y Acad Sci. 2009;1179:216–28. 10.1111/j.1749-6632.2009.05012.x . 19906242

[19] Spijker AT, van Rossum EF. Glucocorticoid receptor polymorphisms in major depression. Focus on glucocorticoid sensitivity and neurocognitive functioning. Ann N Y Acad Sci. 2009;1179:199–215. 10.1111/j.1749-6632.2009.04985.x . 19906241

[20] Zimmermann CA, Arloth J, Santarelli S, Loschner A, Weber P, Schmidt MV, et al Stress dynamically regulates co-expression networks of glucocorticoid receptor-dependent MDD and SCZ risk genes. Transl Psychiatry. 2019;9(1):41 10.1038/s41398-019-0373-1 30696808 PMC6351530

[21] Murrough JW, Iacoviello B, Neumeister A, Charney DS, Iosifescu DV. Cognitive dysfunction in depression: neurocircuitry and new therapeutic strategies. Neurobiol Learn Mem. 2011;96(4):553–63. 10.1016/j.nlm.2011.06.006 . 21704176

[22] Sumiyoshi T, Watanabe K, Noto S, Sakamoto S, Moriguchi Y, Tan KHX, et al Relationship of cognitive impairment with depressive symptoms and psychosocial function in patients with major depressive disorder: Cross-sectional analysis of baseline data from PERFORM-J. J Affect Disord. 2019;258:172–8. 10.1016/j.jad.2019.07.064 . 31426015

[23] Keller AS, Ball TM, Williams LM. Deep phenotyping of attention impairments and the 'Inattention Biotype' in Major Depressive Disorder. Psychol Med. 2019:1–10. 10.1017/S0033291719002290 . 31477195 PMC8022888

[24] Gasbarri A, Sulli A, Packard MG. The dopaminergic mesencephalic projections to the hippocampal formation in the rat. Prog Neuropsychopharmacol Biol Psychiatry. 1997;21(1):1–22. 10.1016/s0278-5846(96)00157-1 . 9075256

[25] Lisman JE, Grace AA. The hippocampal-VTA loop: controlling the entry of information into long-term memory. Neuron. 2005;46(5):703–13. 10.1016/j.neuron.2005.05.002 . 15924857

[26] Shohamy D, Adcock RA. Dopamine and adaptive memory. Trends Cogn Sci. 2010;14(10):464–72. 10.1016/j.tics.2010.08.002 . 20829095

[27] Boku S, Nakagawa S, Toda H, Hishimoto A. Neural basis of major depressive disorder: Beyond monoamine hypothesis. Psychiatry Clin Neurosci. 2018;72(1):3–12. 10.1111/pcn.12604 . 28926161

[28] MacQueen GM, Campbell S, McEwen BS, Macdonald K, Amano S, Joffe RT, et al Course of illness, hippocampal function, and hippocampal volume in major depression. Proc Natl Acad Sci U S A. 2003;100(3):1387–92. 10.1073/pnas.0337481100 12552118 PMC298782

[29] Warner-Schmidt JL, Duman RS. Hippocampal neurogenesis: opposing effects of stress and antidepressant treatment. Hippocampus. 2006;16(3):239–49. 10.1002/hipo.20156 . 16425236

[30] Park SC. Neurogenesis and antidepressant action. Cell Tissue Res. 2019;377(1):95–106. 10.1007/s00441-019-03043-5 . 31165247

[31] Culpepper L. Neuroanatomy and physiology of cognition. J Clin Psychiatry. 2015;76(7):e900 10.4088/JCP.13086tx3c . 26231020

[32] Dillon DG, Pizzagalli DA. Mechanisms of Memory Disruption in Depression. Trends Neurosci. 2018;41(3):137–49. 10.1016/j.tins.2017.12.006 29331265 PMC5835184

[33] Hermans D, Van den Broeck K, Belis G, Raes F, Pieters G, Eelen P. Trauma and autobiographical memory specificity in depressed inpatients. Behav Res Ther. 2004;42(7):775–89. 10.1016/S0005-7967(03)00197-9 . 15149898

[34] Hermans D, Vandromme H, Debeer E, Raes F, Demyttenaere K, Brunfaut E, et al Overgeneral autobiographical memory predicts diagnostic status in depression. Behav Res Ther. 2008;46(5):668–77. 10.1016/j.brat.2008.01.018 . 18342835

[35] Raes F, Hermans D, Williams JM, Beyers W, Brunfaut E, Eelen P. Reduced autobiographical memory specificity and rumination in predicting the course of depression. J Abnorm Psychol. 2006;115(4):699–704. 10.1037/0021-843X.115.4.699 . 17100527

[36] Williams JM, Barnhofer T, Crane C, Herman D, Raes F, Watkins E, et al Autobiographical memory specificity and emotional disorder. Psychol Bull. 2007;133(1):122–48. 10.1037/0033-2909.133.1.122 17201573 PMC2834574

[37] Tye KM, Mirzabekov JJ, Warden MR, Ferenczi EA, Tsai HC, Finkelstein J, et al Dopamine neurons modulate neural encoding and expression of depression-related behaviour. Nature. 2013;493(7433):537–41. 10.1038/nature11740 23235822 PMC4160519

[38] Dubrovina NI. Effects of activation of D1 dopamine receptors on extinction of a conditioned passive avoidance reflex and amnesia in aggressive and submissive mice. Neurosci Behav Physiol. 2006;36(6):679–84. 10.1007/s11055-006-0073-1 . 16783522

[39] El-Ghundi M, O'Dowd BF, George SR. Insights into the role of dopamine receptor systems in learning and memory. Rev Neurosci. 2007;18(1):37–66. 10.1515/revneuro.2007.18.1.37 . 17405450

[40] Fremeau RT Jr., Duncan GE, Fornaretto MG, Dearry A, Gingrich JA, Breese GR, et al Localization of D1 dopamine receptor mRNA in brain supports a role in cognitive, affective, and neuroendocrine aspects of dopaminergic neurotransmission. Proc Natl Acad Sci U S A. 1991;88(9):3772–6. 10.1073/pnas.88.9.3772 2023928 PMC51535

[41] Granado N, Ortiz O, Suarez LM, Martin ED, Cena V, Solis JM, et al D1 but not D5 dopamine receptors are critical for LTP, spatial learning, and LTP-Induced arc and zif268 expression in the hippocampus. Cereb Cortex. 2008;18(1):1–12. 10.1093/cercor/bhm026 . 17395606

[42] Khan ZU, Gutierrez A, Martin R, Penafiel A, Rivera A, de la Calle A. Dopamine D5 receptors of rat and human brain. Neuroscience. 2000;100(4):689–99. 10.1016/s0306-4522(00)00274-8 . 11036203

[43] Tran AH, Tamura R, Uwano T, Kobayashi T, Katsuki M, Ono T. Dopamine D1 receptors involved in locomotor activity and accumbens neural responses to prediction of reward associated with place. Proc Natl Acad Sci U S A. 2005;102(6):2117–22. 10.1073/pnas.0409726102 15684065 PMC548585

[44] Wang Y, Wu J, Zhu B, Li C, Cai JX. Dopamine D1 receptors are responsible for stress-induced emotional memory deficit in mice. Stress. 2012;15(2):237–42. 10.3109/10253890.2011.607525 . 21875304

[45] Weiner DM, Levey AI, Sunahara RK, Niznik HB, O'Dowd BF, Seeman P, et al D1 and D2 dopamine receptor mRNA in rat brain. Proc Natl Acad Sci U S A. 1991;88(5):1859–63. 10.1073/pnas.88.5.1859 1825729 PMC51125

[46] Reichardt HM, Kaestner KH, Tuckermann J, Kretz O, Wessely O, Bock R, et al DNA binding of the glucocorticoid receptor is not essential for survival. Cell. 1998;93(4):531–41. 10.1016/s0092-8674(00)81183-6 . 9604929

[47] Watson LC, Kuchenbecker KM, Schiller BJ, Gross JD, Pufall MA, Yamamoto KR. The glucocorticoid receptor dimer interface allosterically transmits sequence-specific DNA signals. Nat Struct Mol Biol. 2013;20(7):876–83. 10.1038/nsmb.2595 23728292 PMC3702670

[48] Mohajeri MH, Madani R, Saini K, Lipp HP, Nitsch RM, Wolfer DP. The impact of genetic background on neurodegeneration and behavior in seizured mice. Genes Brain Behav. 2004;3(4):228–39. 10.1111/j.1601-1848.2004.00073.x . 15248868

[49] Li K, Nakajima M, Ibanez-Tallon I, Heintz N. A Cortical Circuit for Sexually Dimorphic Oxytocin-Dependent Anxiety Behaviors. Cell. 2016;167(1):60–72 e11. 10.1016/j.cell.2016.08.067 27641503 PMC5220951

[50] Marrocco J, Petty GH, Rios MB, Gray JD, Kogan JF, Waters EM, et al A sexually dimorphic pre-stressed translational signature in CA3 pyramidal neurons of BDNF Val66Met mice. Nat Commun. 2017;8(1):808 10.1038/s41467-017-01014-4 28993643 PMC5634406

[51] Darcet F, Mendez-David I, Tritschler L, Gardier AM, Guilloux JP, David DJ. Learning and memory impairments in a neuroendocrine mouse model of anxiety/depression. Front Behav Neurosci. 2014;8:136 10.3389/fnbeh.2014.00136 24822041 PMC4013464

[52] David DJ, Samuels BA, Rainer Q, Wang JW, Marsteller D, Mendez I, et al Neurogenesis-dependent and -independent effects of fluoxetine in an animal model of anxiety/depression. Neuron. 2009;62(4):479–93. 10.1016/j.neuron.2009.04.017 19477151 PMC2759281

[53] Malisch JL, Saltzman W, Gomes FR, Rezende EL, Jeske DR, Garland T Jr. Baseline and stress-induced plasma corticosterone concentrations of mice selectively bred for high voluntary wheel running. Physiol Biochem Zool. 2007;80(1):146–56. 10.1086/508828 . 17160887

[54] Zalewska K, Ong LK, Johnson SJ, Nilsson M, Walker FR. Oral administration of corticosterone at stress-like levels drives microglial but not vascular disturbances post-stroke. Neuroscience. 2017;352:30–8. 10.1016/j.neuroscience.2017.03.005 . 28288898

[55] Zalewska K, Pietrogrande G, Ong LK, Abdolhoseini M, Kluge M, Johnson SJ, et al Sustained administration of corticosterone at stress-like levels after stroke suppressed glial reactivity at sites of thalamic secondary neurodegeneration. Brain Behav Immun. 2018;69:210–22. 10.1016/j.bbi.2017.11.014 . 29162554

[56] Callaerts-Vegh Z, Beckers T, Ball SM, Baeyens F, Callaerts PF, Cryan JF, et al Concomitant deficits in working memory and fear extinction are functionally dissociated from reduced anxiety in metabotropic glutamate receptor 7-deficient mice. J Neurosci. 2006;26(24):6573–82. 10.1523/JNEUROSCI.1497-06.2006 . 16775145 PMC6674050

[57] Naert A, Callaerts-Vegh Z, D'Hooge R. Nocturnal hyperactivity, increased social novelty preference and delayed extinction of fear responses in post-weaning socially isolated mice. Brain Res Bull. 2011;85(6):354–62. 10.1016/j.brainresbull.2011.03.027 . 21501666

[58] Hughes RN. The value of spontaneous alternation behavior (SAB) as a test of retention in pharmacological investigations of memory. Neurosci Biobehav Rev. 2004;28(5):497–505. 10.1016/j.neubiorev.2004.06.006 . 15465137

[59] Kraeuter AK, Guest PC, Sarnyai Z. The Y-Maze for Assessment of Spatial Working and Reference Memory in Mice. Methods Mol Biol. 2019;1916:105–11. 10.1007/978-1-4939-8994-2_10 . 30535688

[60] Hiramatsu M, Sasaki M, Nabeshima T, Kameyama T. Effects of dynorphin A (1–13) on carbon monoxide-induced delayed amnesia in mice. Pharmacol Biochem Behav. 1997;56(1):73–9. 10.1016/S0091-3057(96)00159-1 . 8981612

[61] Sarter M, Bodewitz G, Stephens DN. Attenuation of scopolamine-induced impairment of spontaneous alteration behaviour by antagonist but not inverse agonist and agonist beta-carbolines. Psychopharmacology (Berl). 1988;94(4):491–5. 10.1007/bf00212843 . 2836875

[62] Ito R, Robbins TW, Pennartz CM, Everitt BJ. Functional interaction between the hippocampus and nucleus accumbens shell is necessary for the acquisition of appetitive spatial context conditioning. J Neurosci. 2008;28(27):6950–9. 10.1523/JNEUROSCI.1615-08.2008 18596169 PMC3844800

[63] Tzschentke TM. Measuring reward with the conditioned place preference (CPP) paradigm: update of the last decade. Addict Biol. 2007;12(3–4):227–462. 10.1111/j.1369-1600.2007.00070.x . 17678505

[64] Ferbinteanu J, McDonald RJ. Dorsal/ventral hippocampus, fornix, and conditioned place preference. Hippocampus. 2001;11(2):187–200. 10.1002/hipo.1036 . 11345125

[65] McDonald RJ, White NM. A triple dissociation of memory systems: hippocampus, amygdala, and dorsal striatum. Behav Neurosci. 1993;107(1):3–22. 10.1037//0735-7044.107.1.3 . 8447956

[66] Anagnostaras SG, Gale GD, Fanselow MS. Hippocampus and contextual fear conditioning: recent controversies and advances. Hippocampus. 2001;11(1):8–17. 10.1002/1098-1063(2001)11:1<8::AID-HIPO1015>3.0.CO;2-7 . 11261775

[67] Bast T, Zhang WN, Feldon J. Dorsal hippocampus and classical fear conditioning to tone and context in rats: effects of local NMDA-receptor blockade and stimulation. Hippocampus. 2003;13(6):657–75. 10.1002/hipo.10115 . 12962312

[68] Goddyn H, Leo S, Meert T, D'Hooge R. Differences in behavioural test battery performance between mice with hippocampal and cerebellar lesions. Behav Brain Res. 2006;173(1):138–47. 10.1016/j.bbr.2006.06.016 . 16860407

[69] Lo AC, De Maeyer JH, Vermaercke B, Callaerts-Vegh Z, Schuurkes JA, D'Hooge R. SSP-002392, a new 5-HT4 receptor agonist, dose-dependently reverses scopolamine-induced learning and memory impairments in C57Bl/6 mice. Neuropharmacology. 2014;85:178–89. 10.1016/j.neuropharm.2014.05.013 . 24863046

[70] Winocur G, Bindra D. Effects of additional cues on passive avoidance learning and extinction in rats with hippocampal lesions. Physiol Behav. 1976;17(6):915–20. 10.1016/0031-9384(76)90008-1 . 14677582

[71] Vandesompele J, De Preter K, Pattyn F, Poppe B, Van Roy N, De Paepe A, et al Accurate normalization of real-time quantitative RT-PCR data by geometric averaging of multiple internal control genes. Genome Biol. 2002;3(7):RESEARCH0034 10.1186/gb-2002-3-7-research0034 12184808 PMC126239

[72] Dwivedi Y, Rizavi HS, Pandey GN. Antidepressants reverse corticosterone-mediated decrease in brain-derived neurotrophic factor expression: differential regulation of specific exons by antidepressants and corticosterone. Neuroscience. 2006;139(3):1017–29. 10.1016/j.neuroscience.2005.12.058 16500030 PMC1513636

[73] Jafari M, Seese RR, Babayan AH, Gall CM, Lauterborn JC. Glucocorticoid receptors are localized to dendritic spines and influence local actin signaling. Mol Neurobiol. 2012;46(2):304–15. 10.1007/s12035-012-8288-3 22717988 PMC3973133

[74] Schaaf MJ, de Jong J, de Kloet ER, Vreugdenhil E. Downregulation of BDNF mRNA and protein in the rat hippocampus by corticosterone. Brain Res. 1998;813(1):112–20. 10.1016/s0006-8993(98)01010-5 . 9824681

[75] Smith MA, Makino S, Kvetnansky R, Post RM. Stress and glucocorticoids affect the expression of brain-derived neurotrophic factor and neurotrophin-3 mRNAs in the hippocampus. J Neurosci. 1995;15(3 Pt 1):1768–77. 10.1523/JNEUROSCI.15-03-01768.1995 . 7891134 PMC6578156

[76] Belujon P, Grace AA. Dopamine System Dysregulation in Major Depressive Disorders. Int J Neuropsychopharmacol. 2017;20(12):1036–46. 10.1093/ijnp/pyx056 29106542 PMC5716179

[77] De Bundel D, Gangarossa G, Biever A, Bonnefont X, Valjent E. Cognitive dysfunction, elevated anxiety, and reduced cocaine response in circadian clock-deficient cryptochrome knockout mice. Front Behav Neurosci. 2013;7:152 10.3389/fnbeh.2013.00152 24187535 PMC3807562

[78] Kempadoo KA, Mosharov EV, Choi SJ, Sulzer D, Kandel ER. Dopamine release from the locus coeruleus to the dorsal hippocampus promotes spatial learning and memory. Proc Natl Acad Sci U S A. 2016;113(51):14835–40. 10.1073/pnas.1616515114 27930324 PMC5187750

[79] Papp M, Gruca P, Lason-Tyburkiewicz M, Litwa E, Niemczyk M, Tota-Glowczyk K, et al Dopaminergic mechanisms in memory consolidation and antidepressant reversal of a chronic mild stress-induced cognitive impairment`. Psychopharmacology (Berl). 2017;234(17):2571–85. 10.1007/s00213-017-4651-4 28567697 PMC5548836

[80] Yadid G, Friedman A. Dynamics of the dopaminergic system as a key component to the understanding of depression. Prog Brain Res. 2008;172:265–86. 10.1016/S0079-6123(08)00913-8 . 18772037

[81] Farley SJ, McKay BM, Disterhoft JF, Weiss C. Reevaluating hippocampus-dependent learning in FVB/N mice. Behav Neurosci. 2011;125(6):871–8. 10.1037/a0026033 22122148 PMC3246014

[82] Oitzl MS, de Kloet ER, Joels M, Schmid W, Cole TJ. Spatial learning deficits in mice with a targeted glucocorticoid receptor gene disruption. Eur J Neurosci. 1997;9(11):2284–96. 10.1111/j.1460-9568.1997.tb01646.x . 9464923

[83] Oitzl MS, Reichardt HM, Joels M, de Kloet ER. Point mutation in the mouse glucocorticoid receptor preventing DNA binding impairs spatial memory. Proc Natl Acad Sci U S A. 2001;98(22):12790–5. 10.1073/pnas.231313998 11606764 PMC60132

[84] Miller BH, Schultz LE, Gulati A, Su AI, Pletcher MT. Phenotypic characterization of a genetically diverse panel of mice for behavioral despair and anxiety. PLoS One. 2010;5(12):e14458 10.1371/journal.pone.0014458 21206921 PMC3012073

[85] Ballegeer M, Van Looveren K, Timmermans S, Eggermont M, Vandevyver S, Thery F, et al Glucocorticoid receptor dimers control intestinal STAT1 and TNF-induced inflammation in mice. J Clin Invest. 2018;128(8):3265–79. 10.1172/JCI96636 29746256 PMC6063488

[86] Siopi E, Denizet M, Gabellec MM, de Chaumont F, Olivo-Marin JC, Guilloux JP, et al Anxiety- and Depression-Like States Lead to Pronounced Olfactory Deficits and Impaired Adult Neurogenesis in Mice. J Neurosci. 2016;36(2):518–31. 10.1523/JNEUROSCI.2817-15.2016 26758842 PMC6602024

[87] Surget A, Saxe M, Leman S, Ibarguen-Vargas Y, Chalon S, Griebel G, et al Drug-dependent requirement of hippocampal neurogenesis in a model of depression and of antidepressant reversal. Biol Psychiatry. 2008;64(4):293–301. 10.1016/j.biopsych.2008.02.022 . 18406399

[88] Judd LL, Schettler PJ, Brown ES, Wolkowitz OM, Sternberg EM, Bender BG, et al Adverse consequences of glucocorticoid medication: psychological, cognitive, and behavioral effects. Am J Psychiatry. 2014;171(10):1045–51. 10.1176/appi.ajp.2014.13091264 . 25272344

[89] Belanoff JK, Gross K, Yager A, Schatzberg AF. Corticosteroids and cognition. J Psychiatr Res. 2001;35(3):127–45. 10.1016/s0022-3956(01)00018-8 . 11461709

[90] McEwen BS, Weiss JM, Schwartz LS. Selective retention of corticosterone by limbic structures in rat brain. Nature. 1968;220(5170):911–2. 10.1038/220911a0 . 4301849

[91] McEwen BS, Weiss JM, Schwartz LS. Uptake of corticosterone by rat brain and its concentration by certain limbic structures. Brain Res. 1969;16(1):227–41. 10.1016/0006-8993(69)90096-1 . 5348850

[92] Conrad CD. A critical review of chronic stress effects on spatial learning and memory. Prog Neuropsychopharmacol Biol Psychiatry. 2010;34(5):742–55. 10.1016/j.pnpbp.2009.11.003 . 19903505

[93] Ibarguen-Vargas Y, Surget A, Touma C, Palme R, Belzung C. Multifaceted strain-specific effects in a mouse model of depression and of antidepressant reversal. Psychoneuroendocrinology. 2008;33(10):1357–68. 10.1016/j.psyneuen.2008.07.010 . 18790573

[94] Mozhui K, Karlsson RM, Kash TL, Ihne J, Norcross M, Patel S, et al Strain differences in stress responsivity are associated with divergent amygdala gene expression and glutamate-mediated neuronal excitability. J Neurosci. 2010;30(15):5357–67. 10.1523/JNEUROSCI.5017-09.2010 20392957 PMC2866495

[95] Hurtubise JL, Howland JG. Effects of stress on behavioral flexibility in rodents. Neuroscience. 2017;345:176–92. 10.1016/j.neuroscience.2016.04.007 . 27066767

[96] Park M, Kim CH, Jo S, Kim EJ, Rhim H, Lee CJ, et al Chronic Stress Alters Spatial Representation and Bursting Patterns of Place Cells in Behaving Mice. Sci Rep. 2015;5:16235 10.1038/srep16235 26548337 PMC4637823

[97] Ogawa T, Okihara H, Kokai S, Abe Y, Karin Harumi UK, Makiguchi M, et al Nasal obstruction during adolescence induces memory/learning impairments associated with BDNF/TrkB signaling pathway hypofunction and high corticosterone levels. J Neurosci Res. 2018;96(6):1056–65. 10.1002/jnr.24216 . 29392750

[98] Salehi A, Rabiei Z, Setorki M. Effect of gallic acid on chronic restraint stress-induced anxiety and memory loss in male BALB/c mice. Iran J Basic Med Sci. 2018;21(12):1232–7. 10.22038/ijbms.2018.31230.7523 30627366 PMC6312671

[99] Allen AP, Curran EA, Duggan A, Cryan JF, Chorcorain AN, Dinan TG, et al A systematic review of the psychobiological burden of informal caregiving for patients with dementia: Focus on cognitive and biological markers of chronic stress. Neurosci Biobehav Rev. 2017;73:123–64. 10.1016/j.neubiorev.2016.12.006 . 27986469

[100] Campeau S, Liberzon I, Morilak D, Ressler K. Stress modulation of cognitive and affective processes. Stress. 2011;14(5):503–19. 10.3109/10253890.2011.596864 21790481 PMC3313908

[101] Girotti M, Adler SM, Bulin SE, Fucich EA, Paredes D, Morilak DA. Prefrontal cortex executive processes affected by stress in health and disease. Prog Neuropsychopharmacol Biol Psychiatry. 2018;85:161–79. 10.1016/j.pnpbp.2017.07.004 28690203 PMC5756532

[102] Coburn-Litvak PS, Pothakos K, Tata DA, McCloskey DP, Anderson BJ. Chronic administration of corticosterone impairs spatial reference memory before spatial working memory in rats. Neurobiol Learn Mem. 2003;80(1):11–23. 10.1016/s1074-7427(03)00019-4 . 12737930

[103] Graybeal C, Feyder M, Schulman E, Saksida LM, Bussey TJ, Brigman JL, et al Paradoxical reversal learning enhancement by stress or prefrontal cortical damage: rescue with BDNF. Nat Neurosci. 2011;14(12):1507–9. 10.1038/nn.2954 22057192 PMC3389817

[104] Monteiro S, Roque S, de Sa-Calcada D, Sousa N, Correia-Neves M, Cerqueira JJ. An efficient chronic unpredictable stress protocol to induce stress-related responses in C57BL/6 mice. Front Psychiatry. 2015;6:6 10.3389/fpsyt.2015.00006 25698978 PMC4313595

[105] van Boxelaere M, Clements J, Callaerts P, D'Hooge R, Callaerts-Vegh Z. Unpredictable chronic mild stress differentially impairs social and contextual discrimination learning in two inbred mouse strains. PLoS One. 2017;12(11):e0188537 10.1371/journal.pone.0188537 29166674 PMC5699833

[106] Sukoff Rizzo SJ, Silverman JL. Methodological Considerations for Optimizing and Validating Behavioral Assays. Curr Protoc Mouse Biol. 2016;6(4):364–79. 10.1002/cpmo.17 27906464 PMC6054129

[107] Willis EF, Bartlett PF, Vukovic J. Protocol for Short- and Longer-term Spatial Learning and Memory in Mice. Front Behav Neurosci. 2017;11:197 10.3389/fnbeh.2017.00197 29089878 PMC5651027

[108] McIlwain KL, Merriweather MY, Yuva-Paylor LA, Paylor R. The use of behavioral test batteries: effects of training history. Physiol Behav. 2001;73(5):705–17. 10.1016/s0031-9384(01)00528-5 . 11566205

[109] Mehla J, Lacoursiere SG, Lapointe V, McNaughton BL, Sutherland RJ, McDonald RJ, et al Age-dependent behavioral and biochemical characterization of single APP knock-in mouse (APP(NL-G-F/NL-G-F)) model of Alzheimer's disease. Neurobiol Aging. 2019;75:25–37. 10.1016/j.neurobiolaging.2018.10.026 . 30508733

[110] Datson NA, Morsink MC, Meijer OC, de Kloet ER. Central corticosteroid actions: Search for gene targets. Eur J Pharmacol. 2008;583(2–3):272–89. 10.1016/j.ejphar.2007.11.070 . 18295201

[111] Binder EB. The role of FKBP5, a co-chaperone of the glucocorticoid receptor in the pathogenesis and therapy of affective and anxiety disorders. Psychoneuroendocrinology. 2009;34 Suppl 1:S186–95. 10.1016/j.psyneuen.2009.05.021 . 19560279

[112] Miranda M, Morici JF, Zanoni MB, Bekinschtein P. Brain-Derived Neurotrophic Factor: A Key Molecule for Memory in the Healthy and the Pathological Brain. Front Cell Neurosci. 2019;13:363 10.3389/fncel.2019.00363 31440144 PMC6692714

[113] Hersi AI, Kitaichi K, Srivastava LK, Gaudreau P, Quirion R. Dopamine D-5 receptor modulates hippocampal acetylcholine release. Brain Res Mol Brain Res. 2000;76(2):336–40. 10.1016/s0169-328x(00)00015-2 . 10762709

[114] Furini CR, Myskiw JC, Schmidt BE, Marcondes LA, Izquierdo I. D1 and D5 dopamine receptors participate on the consolidation of two different memories. Behav Brain Res. 2014;271:212–7. 10.1016/j.bbr.2014.06.027 . 24959860

[115] McNamara CG, Dupret D. Two sources of dopamine for the hippocampus. Trends Neurosci. 2017;40(7):383–4. 10.1016/j.tins.2017.05.005 28511793 PMC5489110

