# Supplementary figures and images for: Cognitive dysfunction in mice lacking proper glucocorticoid receptor dimerization
Source: PLoS One. 2019 Dec 23;14(12):e0226753. doi: 10.1371/journal.pone.0226753 (PMC6927629; doi:10.1371/journal.pone.0226753)

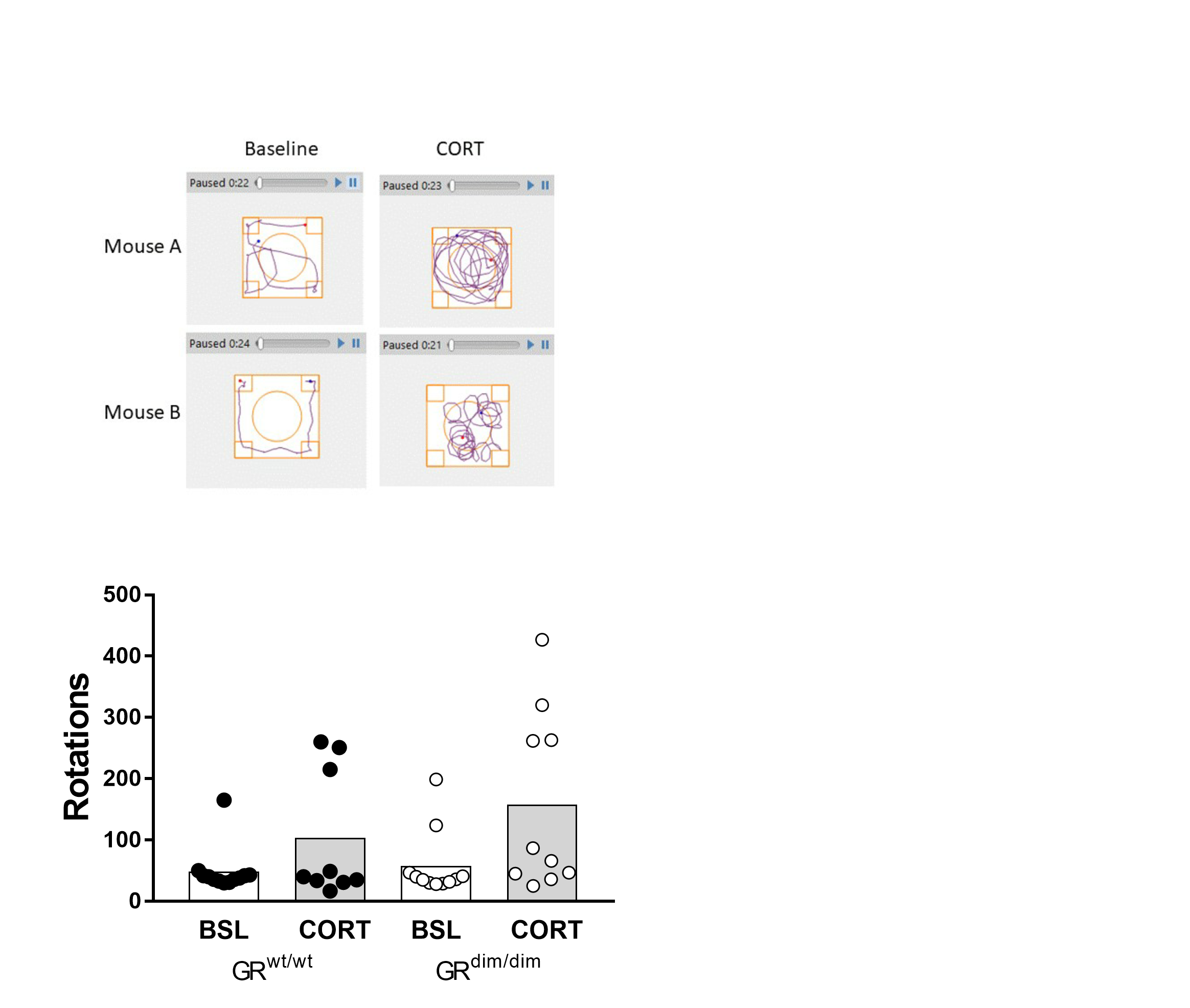

Supplement: S1 Fig — Two examples of increased rotational behaviour after CORT treatment. The two panels on the left show respective tracks for the first 20s of the open field experiment in two mice (A and B) under baseline conditions. Under CORT, the behaviour changes dramatically to fast running in circles in the same mice. (TIF) [file pone.0226753.s001.tif]

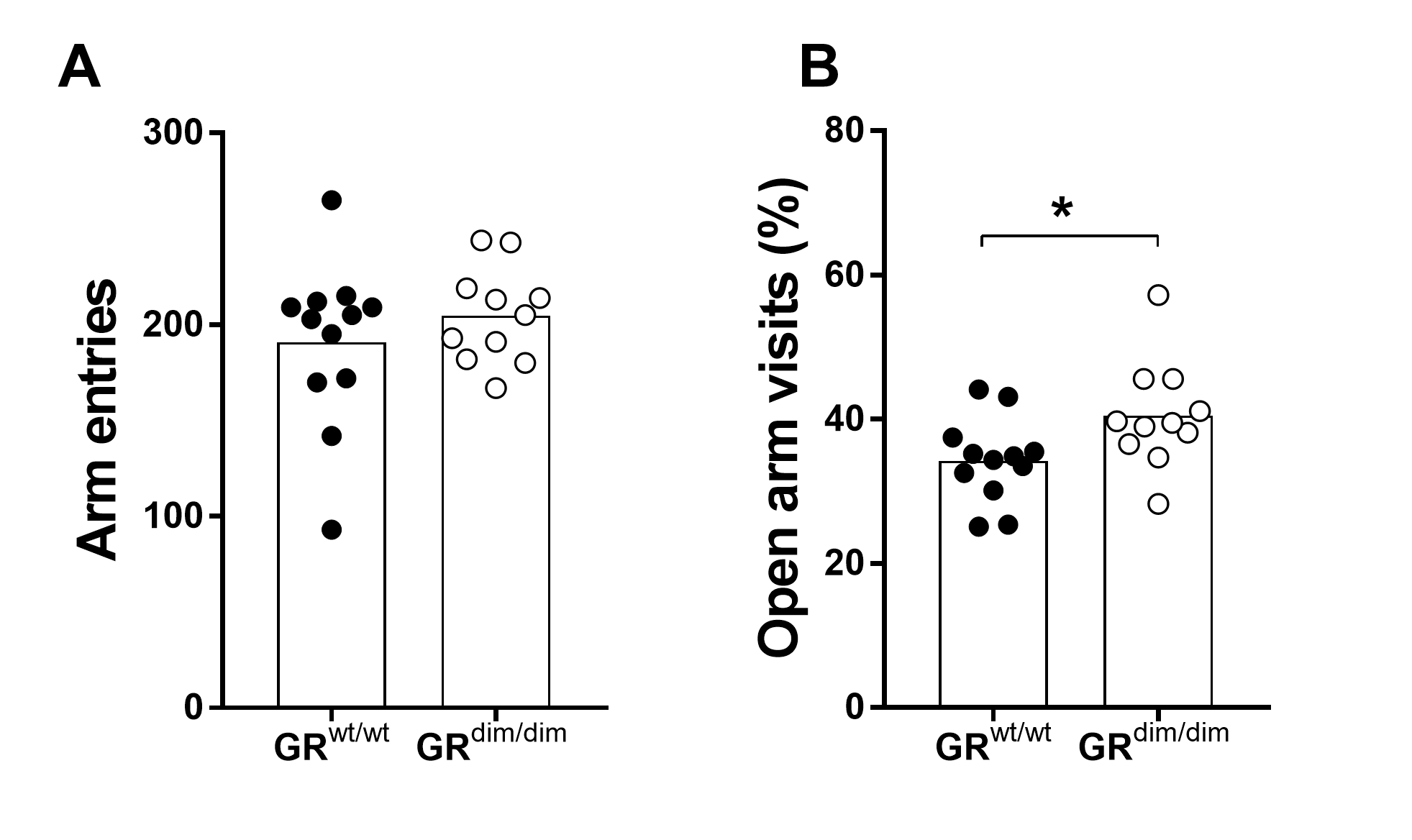

Supplement: S2 Fig — GRdim/dim mice crossed into open arms significantly more, relative to the closed arm, when compared to controls (B). In conjunction with similar general activity [total crosses) (A), indicates that GRdim/dim mice are less anxious than WT controls. Data are presented as mean +/- SEM. * denotes p < 0.05 between genotypes. (TIF) [file pone.0226753.s002.tif]
